# Facial alveolar bone thickness and modifying factors of anterior maxillary teeth: a systematic review and meta-analysis of cone-beam computed tomography studies

**DOI:** 10.1186/s12903-021-01495-2

**Published:** 2021-03-22

**Authors:** Julio Rojo-Sanchis, David Soto-Peñaloza, David Peñarrocha-Oltra, Miguel Peñarrocha-Diago, José Viña-Almunia

**Affiliations:** Oral Surgery Unit, Department of Stomatology, Faculty of Medicine and Dentistry, University of Valencia, Gascó Oliag 1, Valencia, 46010 Spain

**Keywords:** Buccal bone, Facial alveolar bone, Cone-beam computed tomography, CEJ, Maxillary teeth, Meta-analysis

## Abstract

**Background:**

Understanding the anatomy of the facial alveolar bone (FAB), provides a prognostic tool for estimating the degree of dimensional ridge alterations after tooth extraction. This systematic review and meta-analysis aims to determine the FAB thickness and modifying factors of anterior maxillary teeth measured by CBCT scans. A secondary objective was to assess the facial distance from the cementoenamel junction (CEJ) to the bone crest.

**Methods:**

An electronic search was made of Medline, Embase, Web of Science, Cochrane Library and Google Scholar up to December 2019. Studies that analyze and quantitatively compare FAB thickness at maxillary teeth by CBCT scans were included. The methodological quality of the included studies was appraised using the ROBINS-I tool and the overall meta-evidence certainty using the GRADE approach. A single means random-effects meta-analysis was performed to obtain the weighted mean for 95% confidence interval. A meta-regression of covariates and subgroup analysis was conducted. The nullity Q_h_ test and I^2^ index for heterogeneity was estimated.

**Results:**

2560 potentially relevant articles were recorded from which 29 studies were selected for the qualitative analysis, including 17,321 teeth. Seventeen studies considered the facial bone crest, and 12 the CEJ as a reference point for their measurements. Mean FAB thickness was ≤ 1 mm in maxillary incisors and canines (0.75–1.05 mm) and 1–2 mm in premolars. Patients over 50 years of age, females and thin gingival phenotype was associated with thinner FAB at some apico-coronal locations of maxillary incisors and canines. The geographical setting was an effect modifier that could explain up to 87% of the heterogeneity in FAB thickness, being Asian populations that showed the lowest FAB thickness values. The CEJ-bone crest distance was 2–2.5 mm in all teeth analyzed. Population over 50 years of age exhibited greater CEJ-bone crest distances, and males also showed a trend for greater distance. Evidence certainty has shown moderate quality in most analysis subsets.

**Conclusions:**

Facial alveolar bone at anterior maxillary teeth is thin, heterogeneous in width along its apico-coronal dimensions, and increases in thickness in maxillary premolars. The CEJ-bone crest distance presented homogeneous and similar values in all teeth analyzed.

**Supplementary Information:**

The online version contains supplementary material available at 10.1186/s12903-021-01495-2.

## Background

In a large percentage of cases, the anterior maxillary region exhibits very thin facial alveolar bone (FAB), which is often made up only of bundle bone [1]. It is considered to be part of the periodontium, and is coupled to the existence of a tooth root and a periodontal ligament; as a result, it is reabsorbed after tooth extraction [2].

Understanding the anatomy of the alveolar ridges and FAB thickness provides the clinician with a prognostic tool for estimating the degree of future bone loss after tooth extraction [3]. After exodontia, variable amounts of bone resorption occur secondary to qualitative and quantitative changes at the edentulous site of the alveolar process [4, 5]. It is also well known that bone resorption will be greater at the buccal aspect than at the lingual/palatal aspect [2], and that the healthy neighboring dentition maintains bone resorption in proximal areas [6, 7]. The thickness of the FAB is of utmost relevance in the morphological changes of the postextraction alveolus [8]. In this regard, it has been demonstrated that when the thickness is < 1 mm, a mean loss in the height of 7.5 mm occurs after single tooth extraction, while in the cases of thickness ≥ 1 mm, the mean loss in height is 1.1 mm [9]. Moreover, some authors decide the timing of implant placement after single tooth extraction according to FAB thickness [10]. There is currently no consensus regarding the minimal FAB thickness required to avoid ridge resorption [11].

The effect of systemic diseases, occlusal relationship and smoking habits on FAB thickness have been evaluated, but none of these factors has demonstrated a significant influence on FAB anatomy [12]. Zhang et al. [13] in a case–control study showed that postmenopausal women exhibited thinner FAB than premenopausal women and older men. Thus, according to this study, patient hormonal status could influence the facial bone thickness. Some recent studies [14–16] have observed a reduced FAB thickness in individuals aged 50 years or older; patient age therefore also seems to influence FAB thickness.

It has not been determined whether FAB thickness increases or decreases from coronal to apical areas. While some authors have reported an increase in thickness from coronal to apical levels [17–19], others [20, 21] have obtained opposite results, i.e., thicker FAB at the coronal crest. In recent years, several authors [22–25] have studied and analyzed FAB thickness, though much uncertainty remains regarding the different patterns of FAB thickness and the factors that can influence them.

To our knowledge, no previous systematic review has investigated the anatomy of FAB at maxillary teeth. The present systematic review was therefore carried out to critically and comprehensively analyze the FAB thickness of anterior maxillary teeth measured by cone-beam computed tomography (CBCT) scans. A secondary aim was to assess the distance from the cementoenamel junction (CEJ) to the facial bone crest (FBC).

## Methods

This systematic review was conducted following the Preferred Reporting Items for Systematic Reviews and Meta-analyses (PRISMA) [26]. The review was prospectively developed and registered in the PROSPERO database of the University of York, with protocol number: CRD42019120631.

### Focused question

Two a priori defined focused questions based on the participant (P), exposure (E) and outcome (O) format were formulated:

- Q1: *What is the facial alveolar bone thickness at anterior maxillary teeth measured with cone-beam computer tomography scans?*
- Q2: *What is the CEJ-facial bone crest distance at anterior maxillary teeth measured with cone-beam computer tomography scans?*
  - *Population:* Patients with anterior maxillary teeth (from second to the second premolar).
  - *Exposure:* Cone-beam computed tomography scan.
  - *Outcomes:* (i) Facial alveolar bone (FAB) thickness at different apico-coronal levels, measured from facial bone crest (FBC) or CEJ. (ii) The distance from facial bone crest (FBC) to the CEJ.

### Information sources and electronic searching

Two independent reviewers performed an electronic and manual search (JRS and DSP) consulting four main databases and Google Scholar up to December 2019: Medline via PubMed, Web of Science, Embase and Cochrane Library and gray literature. The search strategy combined “MESH” medical subject headings (PubMed) and EMTREE (EMBASE) indexed terms and other free-text terms were combined whenever possible to lessen the risk for selection bias. On a complementary basis, primary source journals related to the study topic were consulted manually covering the last two years. The really syndication service (RSS) feed for PubMed was employed to identify and retrieve new indexed titles fitted to the search strategy. Finally, the reference lists of included studies were consulted to retrieve potential eligible titles, as suggested by Greenhalgh and Peacock [27]. No restrictions were imposed regarding language or date of publication. Discrepancies of retrieved titles were resolved by discussion with a third advisor (J.V.A.). The search strategies tailored for each database are depicted in Table 1.

**Table 1:** Search strategies used for each database, with the corresponding results covering the period up to December 2019

Main databases			
Database	Search strategy employed	Limits	Dec 24, 2019
Pubmed	((((((“Bicuspid”[Mesh] OR “Tooth”[Mesh] OR “Cuspid”[Mesh] OR premolar OR incisor OR canine OR second premolar OR first premolar OR lateral incisor))) NOT ((“Molar”[Mesh] OR “Root Canal Therapy”[Mesh] OR “Root Canal Preparation”[Mesh] OR “Root Canal Obturation”[Mesh] OR impacted canines OR impacted tooth OR “Tooth, Nonvital”[Mesh] OR “Dental Implants, Single-Tooth”[Mesh])))) AND (“Cone-Beam Computed Tomography”[Mesh] OR CBCT OR cone beam volume CT OR cone beam CT OR cone-beam CT)) AND (((CEJ OR cement enamel junction OR vestibular bone peak OR facial bone crest) OR (facial alveolar bone width OR buccal bone thickness OR buccal cortical OR facial bone OR socket bony walls OR facial bone wall OR buccal alveolar bone)))	All text	757
EMBASE	((‘bicuspid’/exp OR ‘bicuspid’ OR ‘tooth’/exp OR ‘tooth’ OR ‘cuspid’/exp OR ‘cuspid’ OR ‘premolar tooth’/exp OR ‘premolar tooth’ OR ‘incisive’ OR ‘canine tooth’/exp OR ‘canine tooth’ OR ‘second premolar’/exp OR ‘second premolar’ OR ‘first premolar’/exp OR ‘first premolar’ OR ‘lateral incisive’) AND (‘computer tomography’/exp OR ‘computer tomography’) OR ‘cbct’ OR ‘cone beam’ OR ‘cone-beam’ OR ‘cone beam computer tomography’) AND (‘cementoenamel junction’ OR cej OR ‘buccal bone crest’ OR ‘facial bone’/exp OR ‘facial bone’ OR ‘facial alveolar bone width’ OR ‘facial alveolar bone’ OR ‘facial alveolar’ OR ‘buccal bone thickness’ OR ‘buccal bone’ OR ‘buccal cortical’ OR ‘socket bone wall’ OR ‘facial bone wall’)	All text	733
COCHRANE	“bicuspid” OR “cuspid” OR Incisor OR “Tooth” OR premolar tooth OR Incisive OR incisive tooth OR second premolar OR lateral incisive in Title Abstract Keyword AND “Cone Beam Computed Tomography” or “Cone Beam Computer Assisted Tomography” or “Cone Beam Computerized Tomography” or “Cone Beam CT” or “Volumetric Computed Tomography” or “Volumetric CT” or “Cone-Beam Computed Tomography” or “Cone-Beam Computer-Assisted Tomography” or “Cone-Beam Computerized Tomography” or “Cone-Beam CT” or “Cone-Beam CT Scan” in Title Abstract Keyword AND “cement enamel junction” OR CEJ OR Vestibular bone peak OR “facial bone” OR “Cortical Bone” OR Facial alveolar bone width OR buccal bone OR facial alveolar bone wall OR buccal alveolar bone OR buccal bone thickness OR buccal cortical OR facial bone OR facial bone wall in Title Abstract Keyword NOT “root canal therapy” OR “root canal-therapy” OR “root canal obturation” OR “root canal preparation” OR impacted canine OR “impacted tooth” OR “Impacted Teeth” OR “tooth non-vital” OR “Dental Implants, Single Tooth” OR “Dental implant” in Title Abstract Keyword—(Word variations have been searched)	All text	65
WOS	#1 TS = (bicuspid) OR TS = (cuspid) OR TS = (tooth) OR TS = (premolar) OR TS = (incisor) OR TS = (canine) OR TS = (first premolar) OR TS = (second premolar) OR TS = (lateral incisor)	All text	858
	#2 TS = (computed tomography) OR TS = (CBCT) OR TS = (cone beam) OR TS = (cone-beam) OR TS = (cone beam computer tomography)		
	#3 TS = (cement enamel junction OR CEJ) OR TS = (facial bone crest OR vestibular bone peak) OR TS = (facial bone) OR TS = (facial alveolar bone width) OR TS = (facial alveolar bone) OR TS = (facial alveolar) OR TS = (buccal bone thickness) OR TS = (buccal bone) OR TS = (buccal cortical) OR TS = (socket bone wall) OR TS = (facial bone wall)		
	#4 #1 AND #2 AND #3		
	#5 TS = (root canal therapy OR root canal-therapy OR root canal obturation OR root canal preparation) OR TS = ( impacted canine) OR TS = (impacted tooth) OR TS = (Impacted Teeth) OR TS = (tooth non-vital) OR TS = (Dental Implants, Single Tooth) OR TS = (Dental implant)		
	#6 #4 NOT #5		
Scholar	(Premolar OR incisor OR canine OR second premolar OR first premolar OR lateral incisor) AND (“Cone-Beam Computed Tomography”[Mesh] OR CBCT) AND (Cement enamel junction OR CEJ OR Facial bone crest OR facial alveolar bone OR buccal bone)		147
		Total	2560
		Duplicates	557
		After duplicates removal	2003

### Eligibility criteria

Studies were selected according to the following eligibility criteria:

- Inclusion criteria: observational clinical studies (e.g., retrospective cohort, cross-sectional, case–control studied) and randomized controlled trials if any, that analyze and quantitatively compare FAB thickness at anterior maxillary teeth (incisors, canines and premolars), using CEJ or FBC as a tomographic anatomical reference point.
- Exclusion criteria: articles investigating FAB only with calipers, or computed tomography scans limited to mandibular or posterior maxillary teeth; studies presenting a sample size of < 30 patients (as a threshold according to central limit theorem for samples with a normal distribution), included patients with dental absences at the anterior maxillary teeth or included patients under 18 years; studies that included patients under orthodontic treatment, bone augmentation procedures, systemic diseases affecting bone metabolism, case reports, case series, abstracts, interviews, editorials and expert opinions.

### Study selection

The titles and abstracts were screened in duplicate (JRS and DSP) using a predefined Excel spreadsheet (Excel for Mac ver. 16.16.2, Microsoft®, Redmond, WA, USA). In a second stage, the full-text files of potentially eligible titles were reviewed for definitive inclusion. Eligibility agreement between reviewers was assessed through kappa scores (Cohen’s ĸ coefficient) and interpreted using the Landis and Koch scale. Disagreements were resolved by discussion with a third advisor (J.V.A.).

### Study outcomes and evaluation

Study data were extracted by duplicate (JRS and DSP) seeking comparability in predefined Excel spreadsheets (Excel for Mac ver. 16.16.2, Microsoft ®, Redmond, WA, USA). The following items were considered: author/year, country, sample size, study design, study quality, age, sex, teeth analyzed, CBCT purpose, presence of dehiscence or fenestration, CBCT general setting, FAB thickness and CEJ-FBC distance. The main objectives of the present study were:

- *(i) Primary outcome:* FAB thickness at different apico-coronal levels, measured from FBC or CEJ, expressed in mm.
- *(ii) Secondary outcome:* The distance from the CEJ to the FBC, expressed in mm.

**Fig. 1:**
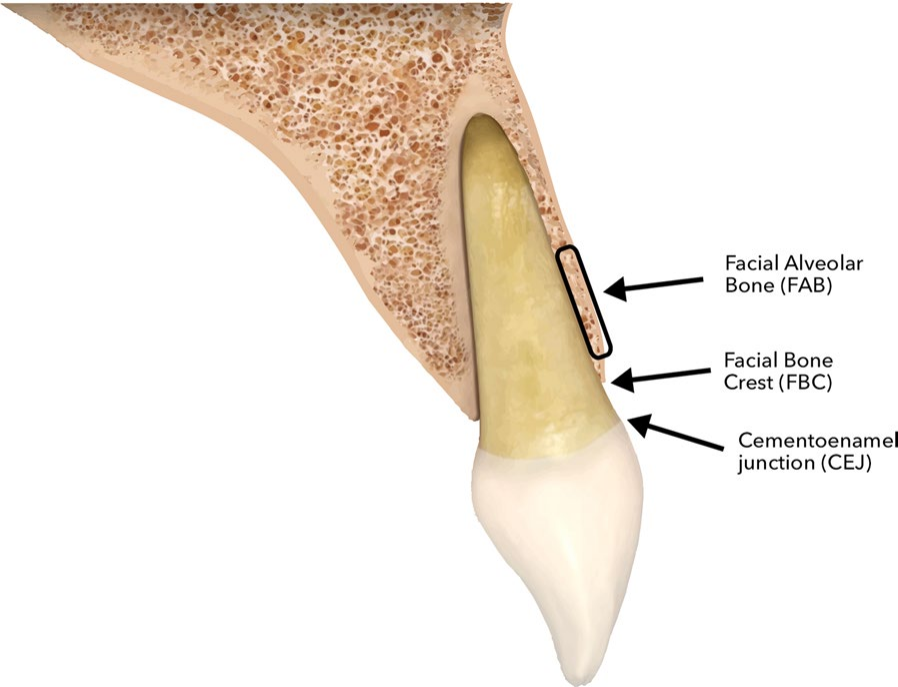
Location of anatomical landmarks: facial alveolar bone (FAB), facial bone crest (FBC) and cementoenamel junction (CEJ)

These outcomes were assessed using different anatomical landmarks (Fig. 1):

- *Cementoenamel junction (CEJ*): The anatomical limit between the anatomical crown and root surface, defined as the junction zone of the cementum and enamel in the cervical region of the tooth [28].
- *Facial bone crest (FBC):* The highest coronal point of facial alveolar bone at the central site of the tooth [29].

### Risk of bias of individual studies

Quality assessment was performed in duplicate by two independent reviewers (J.R.S. and D.S.P.). The ROBINS-I tool from the Cochrane Collaboration was used for nonrandomized studies of interventions (NRSI) [30]. The tool comprises seven items related to pre-intervention (confounding and selection bias), intervention (classification bias) and post-intervention stages (reporting bias). The studies were judged to have a low risk (low risk of bias for all domains), moderate risk (low or moderate risk of bias for all domains), serious risk (serious risk of bias in at least one domain, but not a critical risk of bias in any domain), critical risk (critical risk of bias in at least one domain) or no information (no clear indication that the study is at serious or critical risk of bias and there is a lack of information in one or more key domains of bias).

### Quantitative synthesis and meta-analysis

Quantitative data were summarized in predefined Excel spreadsheets, seeking comparability between studies. The single mean and standard deviation (mean ± SD) of FAB thickness at the different apico-coronal levels were estimated, and the distance from CEJ to FBC at the different teeth of the anterior maxilla was also calculated to explore potential interactions with the FAB values. Concerning the primary study objective, the strategy was to segment the data into two groups according to the reference point employed to measure the thickness of FAB: CEJ or FBC.

If a study failed to provide enough data to estimate the mean and SD of the FAB values, an e-mail was sent to the corresponding author requesting raw data. A meta-analysis of single means was performed. The I^2^ index of heterogeneity and the corresponding nullity statistical Q-test was calculated—I^2^ values of 25%, 50% and 75% being interpreted as indicating low, moderate and high heterogeneity, respectively. The consistency of results was explored through Galbraith plots. Publication bias was investigated by visual detection on the funnel plot and employing the Egger’s test if possible (≥ 10 studies) [31]. A subgroup analysis was designed to assess the effect of age, sex and gingival phenotype.

### Additional analyses

In the case of high heterogeneity, a mixed-effects model meta-regression analysis was performed to detect the effect of potential effect modifiers upon analysis consistency. The impact on heterogeneity being represented by the R-square statistic [32]. A level of significance of 5% (α = 0.05) was established. The R 3.5.1 package (R Foundation for Statistical Computing, Vienna, Austria), was employed to perform the present meta-analysis.

The following variables are considered: geographic setting, propensity for confounding- and selection-bias (moderate to serious concerns), periodontal disease, smoking and voxel size resolution.

- *Periodontal disease:* If the inclusion criteria of primary studies disclose explicitly for no periodontal disease or refer to physiologic bone loss levels accepted as healthy periodontium (1.0–3.0 mm) from CEJ [33].
- *Smoking*: The tobacco consumption increases the risk of osteoporosis [34] and has a deleterious effect in periodontal health status [35].
- *Voxel size resolution:* A voxel size of 0.3 to 0.4 mm is acceptable for diagnostic quality for implant treatment planning [36].

### Evidence certainty

The GRADE approach using the GRADEpro|GDT software (https://gdt.gradepro.org/app/) was used to assess the overall quality of meta‐evidence, according to its level of certainty: very low, low, moderate, or high according to the GRADE handbook (https://gdt.gradepro.org/app/handbook/handbook.html). The following items are incorporated in summary of findings tables (SoF): risk of bias, inconsistency, indirectness, imprecision and other reasons. This approach is based on the metric scale provided by the ROBINS-I tool for risk of bias. Because included studies are observational, these begin the assessment with a certainty reduction by two levels.

To assess evidence by imprecision, in a first instance, the 95% CI is inspected to ascertain that not includes/crossing the zero value (null effect). If the confidence interval tends toward a side (positive or negative) a maximum error measurement of about ± 0.20 mm is established as a threshold for measurement accuracy (as showed in SoF). Meta-analytic standard deviations surpassing in a tenth, the error point, are considered imprecise.

## Results

### Study selection

**Fig. 2:**
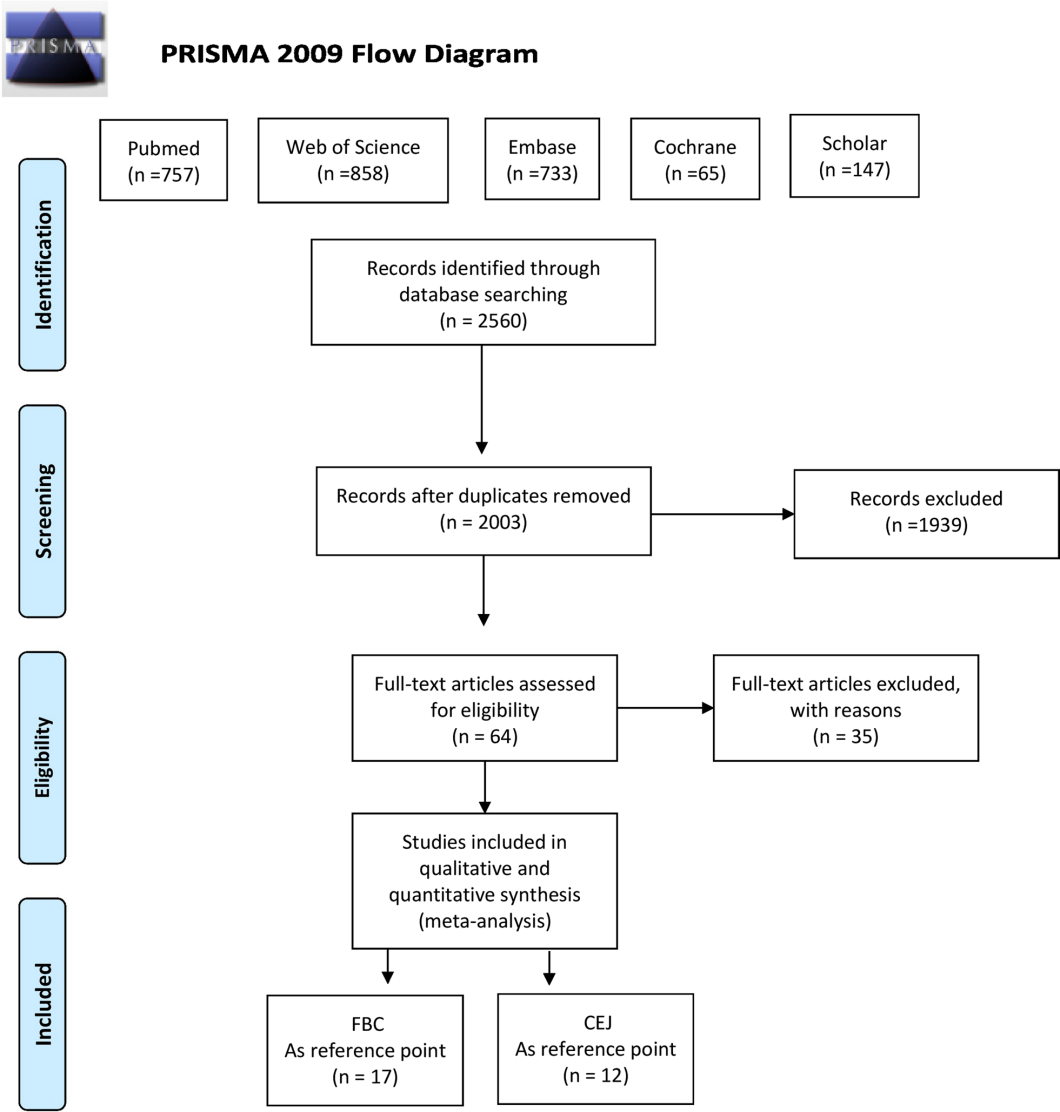
Flow chart of the included studies. *FBC* facial bone crest, *CEJ* cementoenamel junction

A total of 2560 potentially relevant articles were found through sensitive searches (Fig. 2). After the removal of duplicates, a total of 2003 titles and abstracts were considered to be potentially eligible. Two reviewers (J.R.S. and D.S.P.) were calibrated in the application of the inclusion and exclusion criteria. In the second phase, 64 articles were obtained and assessed by reading the full text. For the systematic review, 29 articles fulfilled the inclusion criteria. The reviewers showed almost perfect agreement (k = 0.83). Discussion with a third advisor (J.V.A.) solved discrepancies between the two reviewers. The reasons for study exclusion after full-text appraisal were recorded (Additional file 1).

### Study characteristics

The 29 included studies were published between 2010 and 2019, with the majority being published in the last five years (Additional file 2). Three included studies were retrospective cohort studies, two were case–control studies, and 24 had a cross-sectional design. All of them measured FAB thickness with CBCT performed previously for diagnostic purposes. Most studies were moderate to high in terms of sample size (median of 120 patients per study) and 19 analyzed more than 300 teeth. The 29 included studies comprised 3556 patients and measured 17,321 teeth, including maxillary central incisors (CI), lateral incisors (LI), canines (C), first premolars (1PM) and second premolars (2PM). According to the geographical setting of the study sample, 15 studies were made in populations from Asia, five from America, six from Europe and three from Africa. Sixteen studies [12–17, 19, 23, 24, 37–43] analyzed FAB thickness considering FBC as the anatomical reference point, while 13 studies [20, 22, 25, 44–53] used CEJ. Fifteen studies analyzed the CEJ-FBC distance; ten studies evaluated the prevalence of FAB thickness < 1 mm, and only 7 analyzed the presence of dehiscence or fenestration.

### Risk of bias of individual studies

**Fig. 3:**
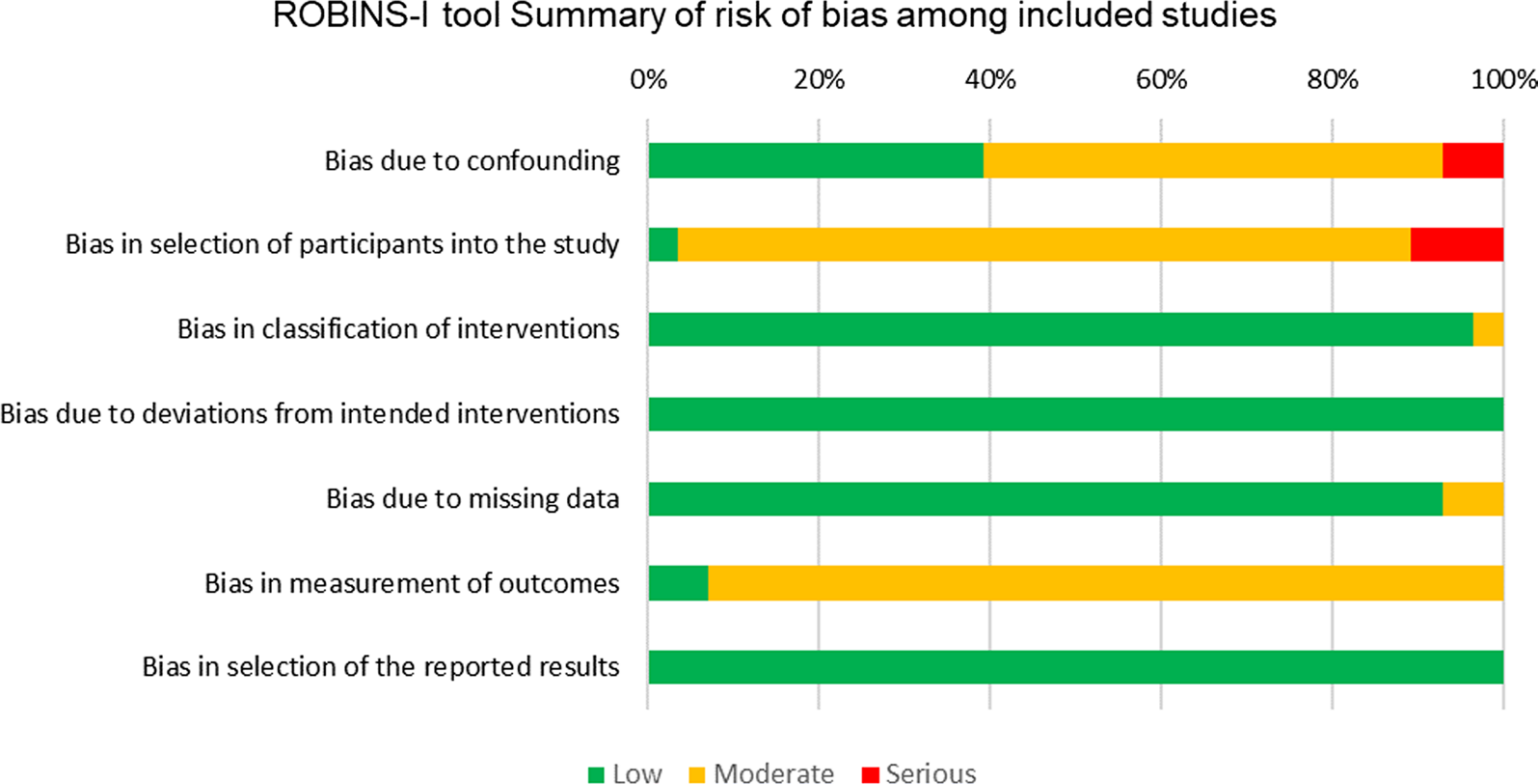
Risk of bias of individual studies analyzed by the ROBINS-I tool. Four studies showed a high risk of bias (10.34%), while the majority (n = 26; 86.95%) showed a moderate risk of bias

Assessment of the risk of bias of the included studies with the ROBINS—I tool, showed that only three studies to have a high risk of bias (10.34%) while the majority of the studies (n = 26; 89.65%) presented a moderate risk of bias (Fig. 3). The most problematic domains were referred to confounding factors (serious in 6.89% of the studies) and in the selection of participants in the study (serious in 10.34% of the studies). Low risk of bias was reported for the classification of interventions, due to deviations from intended interventions, missing data, measurements of outcomes and for selection of the reported results in the majority of the studies. Only 1 study [22], performs a priori power calculation.

### Quantitative synthesis of the results

**Fig. 4:**
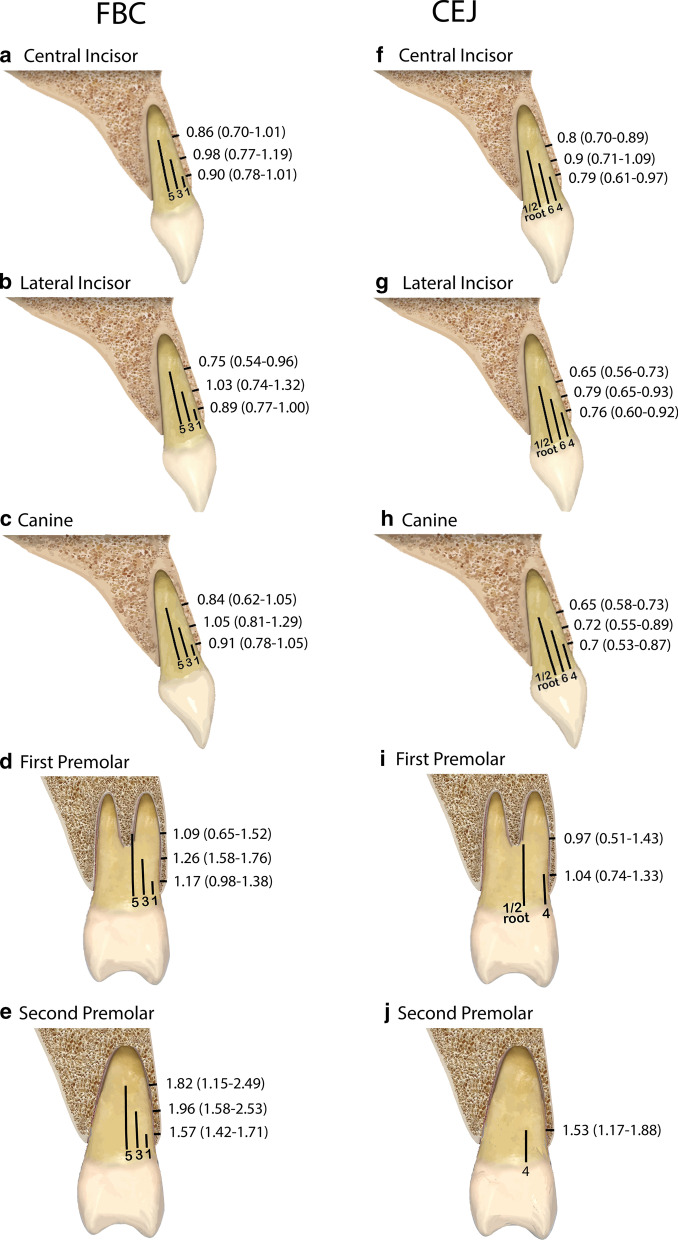
Facial alveolar bone thickness mean values and ranges expressed in mm. The anterior maxillary teeth measured are represented in the following figures: **a**, **f** CI; **b**, **g** LI; **c**, **h** C; **d**, **i** 1PM; **e**, **j** 2PM. FAB thickness taking FBC as the anatomical reference point is represented in **a**–**e**. FAB thickness taking CEJ as the anatomical reference point is represented in figures **f**–**j**

The thickness of FAB was the main outcome of the present review. However, the different anatomical reference points among the studies were the major limitation establishing comparisons. Data from studies in which FBC was the reference point considered for measurement were analyzed independently from studies that deem CEJ as the anatomical reference point (Fig. 4). The sample of each included study was divided into different age groups to evaluate the influence of age on FAB thickness. However, most of the articles had groups over and under 50 years, so this age was used to compare them.

### FAB thickness considering FBC as the anatomical reference point

Seventeen studies (9264 teeth) used FBC as a reference point. The measurements on which most of the articles agreed were at 1, 3 and 5 mm from FBC. FAB was thicker as the analyzed tooth was located in a more posterior area (Table 2). Mean thickness values at anterior teeth (CI, LI and C) were ≤ 1 mm (0.75–1.05). At maxillary premolars (1PM and 2PM) mean FAB thickness was > 1 mm (1.09–1.96). In all teeth analyzed, mean thicker FAB was found at 3 mm when compared with the measurements at 1 and 5 mm from the FBC. These findings are observed in a high heterogeneity context.

**Table 2:** Facial alveolar bone thickness at CI, LI, C, 1PM and 2PM at different points from FBC and CEJ

Tooth type	N (teeth number)	Reference point	WM	SE	95% CI	I^2^	Q_H_ (p-value)	Egger (p-value)	Certainty of the evidence (grade)
*FAB thickness from FBCFAB thickness from FBC*									
CI	14 (3038)	1 mm	0.90	0.06	0.78–1.01	99.30%	< 0.001***	0.332	⨁⨁◯◯LOW
	7 (1369)	3 mm	0.98	0.11	0.77–1.19	99.30%	< 0.001***	–	⨁⨁⨁◯MODERATE
	8 (1654)	5 mm	0.86	0.08	0.70–1.01	98.80%	< 0.001***	–	⨁⨁⨁◯MODERATE
	13 (2721)	1 mm	0.89	0.06	0.77–1.00	98.90%	< 0.001***	0.494	⨁⨁⨁◯MODERATE
LI	6 (957)	3 mm	1.03	0.15	0.74–1.32	99.30%	< 0.001***	–	⨁⨁⨁◯MODERAT
	7 (1266)	5 mm	0.75	0.11	0.54–0.96	99.10%	< 0.001***	–	⨁⨁⨁◯MODERATE
C	9 (1652)	1 mm	0.91	0.07	0.78–1.05	98.70%	< 0.001***	–	⨁⨁⨁◯MODERATE
	6 (1006)	3 mm	1.05	0.12	0.81–1.29	98.80%	< 0.001***	–	⨁⨁⨁◯MODERATE
	6 (1150)	5 mm	0.84	0.11	0.62–1.05	98.80%	< 0.001***	–	⨁⨁⨁◯MODERATE
	4 (491)	1 mm	1.17	0.1	0.98–1.38	95.30%	< 0.001***	–	⨁⨁⨁◯MODERATE
1PM	3 (461)	3 mm	1.26	0.26	0.76–1.76	98.80%	< 0.001***	–	⨁⨁◯◯LOW
	3 (432)	5 mm	1.09	0.22	0.65–1.52	98.50%	< 0.001***	–	⨁⨁◯◯LOW
2PM	4 (534)	1 mm	1.57	0.07	1.42–1.71	83.80%	0.002**	–	⨁⨁⨁◯MODERATE
	3 (508)	3 mm	1.96	0.29	1.38–2.53	98.30%	0.004**	–	⨁⨁◯◯LOW
	3 (508)	5 mm	1.82	0.34	1.15–2.49	98.60%	< 0.001***	–	⨁⨁◯◯LOW
*FAB thickness from CEJ*									
CI	7 (1618)	4 mm	0.79	0.09	0.61–0.97	99.40%	< 0.001***	–	⨁⨁◯◯LOW
	4 (769)	6 mm	0.9	0.09	0.71–1.09	99.00%	< 0.001***	–	⨁⨁◯◯LOW
	7 (1847)	middle root	0.8	0.05	0.70–0.89	97.60%	< 0.001***	–	⨁⨁◯◯LOW
	7 (1606)	4 mm	0.76	0.08	0.60–0.92	98.80%	< 0.001***	–	⨁⨁◯◯LOW
LI	3 (423)	6 mm	0.79	0.07	0.65–0.93	95.80%	< 0.001***	–	⨁⨁⨁◯MODERATE
	6 (1562)	middle root	0.65	0.04	0.56–0.73	96.40%	< 0.001***	–	⨁⨁⨁◯MODERATE
C	7 (1544)	4 mm	0.7	0.09	0.53–0.87	98.70%	< 0.001***	–	⨁⨁◯◯LOW
	3 (368)	6 mm	0.72	0.09	0.55–0.89	96.50%	< 0.001***	–	⨁⨁◯◯LOW
	6 (1555)	middle root	0.65	0.04	0.58–0.73	92.90%	< 0.001***	–	⨁⨁⨁◯MODERATE
1PM	3 (744)	4 mm	1.04	0.15	0.74–1.33	97.90%	< 0.001***	–	⨁⨁◯◯LOW
	2 (680)	middle root	0.97	0.24	0.51–1.43	98.50%	< 0.001***	–	⨁⨁◯◯LOW
2PM	2 (664)	4 mm	1.53	0.18	1.17–1.88	95.00%	< 0.001***	–	⨁⨁◯◯LOW

*N* study number, *WM* weighted mean, *SE* standard error, *I*^2^ I-squared, *CI* confidence interval, *QH* Cochran’s Q^I2^

^*^p < 0.05; **p < 0.01; ***p < 0.001 (Strength of statistical significance)

**Fig. 5:**
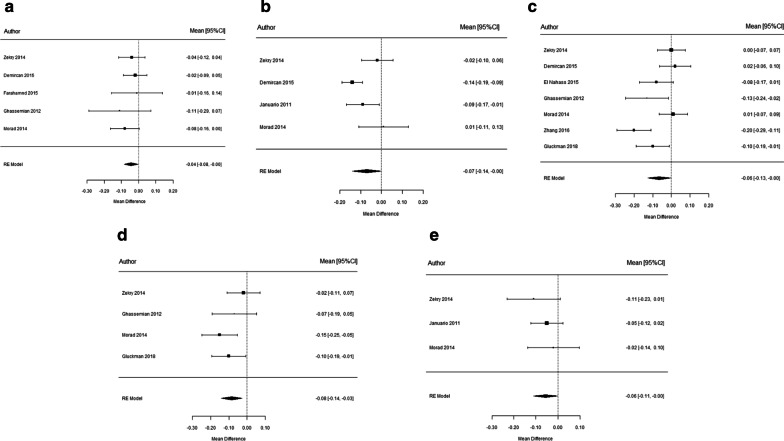
Forest plots of the influence of age and sex upon FAB thickness at CI (**a**, **b**), LI (**c**) and C (**d**, **e**) at different measurement location from FBC

Sub-group analyses were conducted by age and gender. The analysis by age groups showed that at 5 mm from FBC, significantly thinner FAB was observed in > 50 years groups for CI and C (p < 0.05). About gender, significantly thicker FAB in males was found at 1 mm from the FBC in LI and C (p < 0.05), and at 5 mm from FBC in CI (p = 0.034) (Fig. 5). No statistically significant differences were found referred to the other teeth and sites of measurement for age and sex (Additional file 3).

### FAB thickness considering CEJ as the anatomical reference point

Twelve studies (8057 teeth) used CEJ as the anatomical reference point. The measurement locations on which most of the articles agreed were at 4 and 6 mm from CEJ and at the middle root level.

The mean FAB thickness values were < 1 mm in all anterior teeth at all measurement locations (0.65–0.9); also, thicker mean values were found at 6 mm from the CEJ. At maxillary premolars, the mean FAB thickness was > 1 mm, except for 1PM at the middle root (Table 2). These findings were observed in a high heterogeneity context.

Three studies [44, 45, 49] evaluated the influence of gingival phenotype in FAB thickness at CI, LI and C. Significantly thicker FAB was reported at thick gingival phenotype in all anterior teeth and measurement locations (p < 0.05).

### Prevalence of < 1 mm and < 0.5 mm FAB

Ten studies (5516 teeth) evaluated the prevalence of a FAB thickness of less than 1 mm and 0.5 mm. The prevalence of < 1 mm FAB thickness was 69.9% in CI, 64.5% in LI, 55% in C and 40.4% at 1PM; while the prevalence of < 0.5 mm was 30.2% in CI and 35.4% in LI. There were not enough data to evaluate these prevalences at 2PM either the percentage of < 0.5 mm FAB thickness in C and 1PM.

### CEJ-FBC distance

Fifteen studies (12,391 teeth) evaluated CEJ-FBC distance. The mean values were between 2.02 and 2.53 mm (Table 3). Considering the different age subgroups, it was a significantly greater CEJ-FBC distance in patients over 50 years of age referred to CI, LI and C (Table 3). About gender, males showed a greater CEJ-FBC distance in CI (p < 0.001). There was not enough data to assess the variability of age and gender among studies.

**Table 3:** Measurement of CEJ-FBC distance at CI, LI, C, 1PM and 2PM, and analyzed by age and gender subgroups

Tooth type	N (teeth number)	Variables	WM	SE	95%CI	I^2^	Q_H_ (p-value)	Egger (p-value)	z (p-value)	Certainty of the evidence (grade )
CI	14 (3024)	Mean distance	2.24	0.12	2.00–2.48	98.70%	< 0.001***	0.080	< 0.001***	⨁⨁⨁◯MODERATE
	5 (1032)	Sex	− 0.32	0.11	− 0.53 0.12	66.60%	< 0.001***	–	0.002**	⨁⨁⨁◯MODERATE
	6 (1798)	Age	0.83	0.28	0.29 1.38	98.60%	< 0.001***	–	0.003**	⨁⨁⨁◯MODERATE
LI	13 (2685)	Mean distance	2.35	0.11	2.13–2.57	98.40%	< 0001***	0.186	–	⨁⨁⨁◯MODERATE
	4 (722)	Sex	− 0.19	0.14	− 0.46 0.09	73.10%	0.011*	–	0.184	⨁⨁◯◯LOW
	6 (1690)	Age	0.82	0.23	0.37 1.27	97.90%	< 0.001***	–	< 0.001***	⨁⨁⨁◯MODERATE
C	10 (2111)	Mean distance	2.53	0.15	2.24–2.81	98.60%	< 0.001***	–	–	⨁⨁⨁◯MODERATE
	3 (545)	Sex	0.01	0.32	− 0.61 0.63	89.40%	< 0.001***	–	0.969	⨁⨁◯◯LOW
	5 (1513)	Age	0.75	0.24	0.27 1.23	96.60%	< 0001***	–	0.002**	⨁⨁⨁◯MODERATE
1PM	3 (907)	Mean distance	2.33	0.08	2.16–2.49	86.70%	< 0.001***	–		⨁⨁⨁◯MODERATE
2PM	3 (952)	Mean distance	2.02	0.11	1.81–2.22	93.60%	< 0.001***	–	–	⨁⨁⨁◯MODERATE

*N* study number, *WM* weighted mean, *SE* standard error, *CI* confidence interval, *I*^2^ I-squared, *QH* Cochran’s Q

^*^p < 0.05; **p < 0.01; ***p < 0.001 (Strength of statistical significance)

### Dehiscences and fenestrations

Seven studies (4295 teeth) evaluated the presence of bone dehiscences and fenestrations. The prevalence of bone dehiscence was 12.3% in CI, 14.3% in LI and 20.1% in C; while the prevalence of bone fenestration was 6.4% in CI, 21.6% in LI and 23.8% in C. Concerning maxillary premolars, the available data were too limited to evaluate these parameters.

### Publication bias

The test for publication bias was planned in meta-analysis with at least ten studies. The Egger’s test not found hints for publications bias among meta-analyses nor for sub-group analyses assessed (p > 0.05). (Tables 2 and 3).

### Meta-regression analysis

**Fig. 6:**
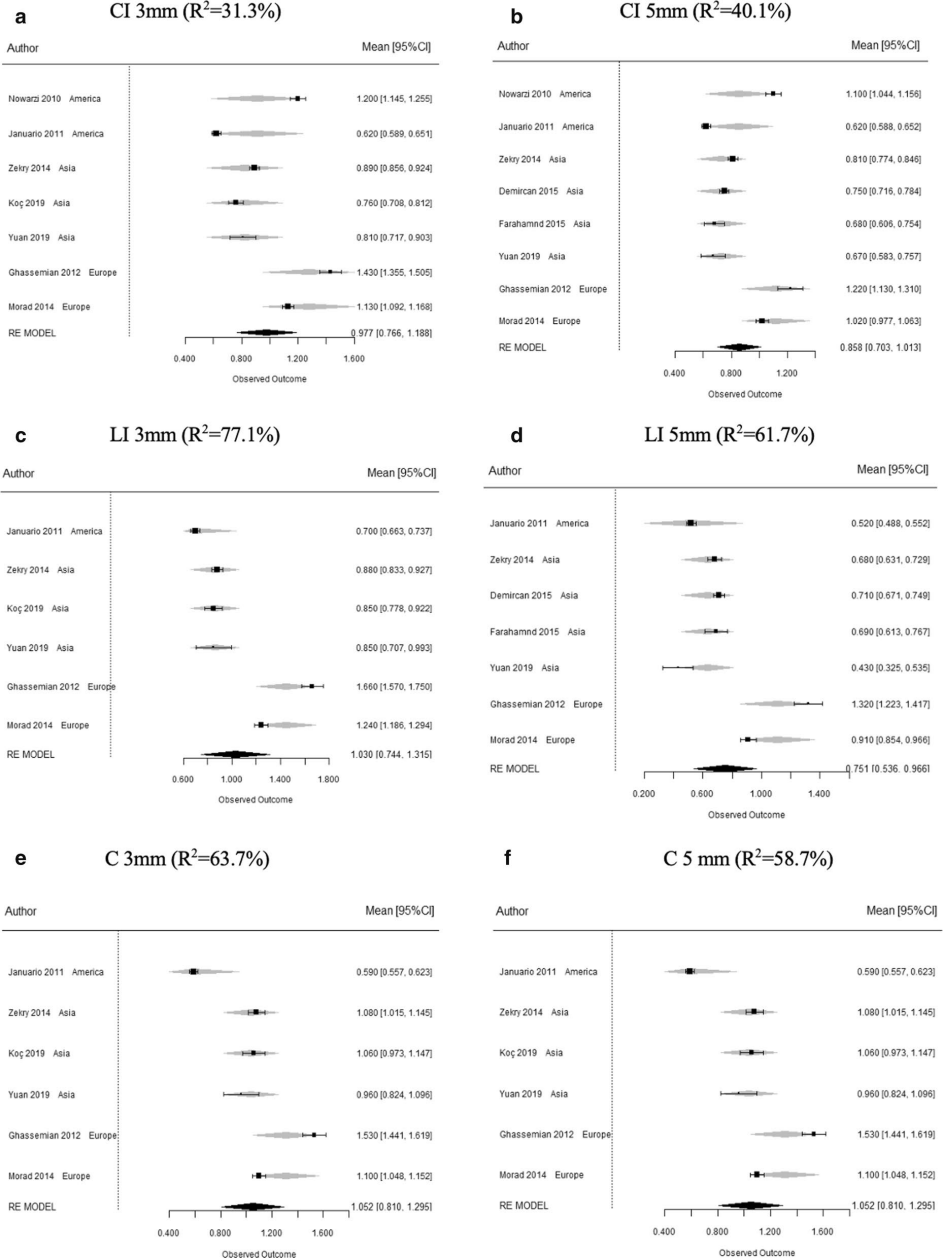
Forest plots of the meta-regression analysis of the geographical setting in FAB thickness of CI (**a**, **b**), LI (**c**, **d**) and C (**e**, **f**) at 3 and 5 mm from FBC

Geographical setting showed a significant effect (p < 0.05) in most measurements as depicted in Table 4. This parameter explains a significant part of the inconsistency at all measurements for concerning LI and C, and at 5 mm in CI. At C, Asian population reported significantly thicker FAB than those from Africa and America at 1 (R2 = 87.1%) and 3 mm (R2 = 63.7%) from the FBC; also at 5 mm from the FBC studies from Europe showed significantly thicker FAB than those from Asia (R2 = 58.71%) (Fig. 6).

**Table 4:** Facial alveolar bone thickness using FBC as reference point, with meta-regression of sample geographical setting at CI, LI and C

Tooth type	Reference point	N (teeth number)	Geographical setting	Beta	SE	95% CI	z (p-value)	R^2^
CI	1 mm	14 (3038)	America (ref.)				0.139	16.00%
			Asia	− 0.06	0.14	− 0.34 0.21	0.645	
			Europe	0.15	0.16	− 0.17 0.47	0.369	
			Africa	− 0.27	0.18	− 0.63 0.08	0.132	
	3 mm	7 (1369)	America (ref.)				0.094	31.30%
			Asia	− 0.08	0.22	− 0.51 0.33	0.682	
			Europe	0.37	0.24	− 0.09 0.83	0.118	
	5 mm	8 (1654)	America (ref.)				0.037*	40.10%
			Asia	− 0.13	0.15	− 0.42 0.16	0.389	
			Europe	0.26	0.17	− 0.08 0.59	0.135	
LI	1 mm	13 (1726)	America (ref.)				0.049*	29.30%
			Asia	− 0.14	0.14	− 0.42 0.14	0.322	
			Europe	0.16	0.16	− 0.16 0.47	0.336	
			Africa	− 0.24	0.17	− 0.59 0.11	0.174	
	3 mm	6 (957)	Asia (ref.)				< 0.001***	77.10%
			Europe	0.58	0.16	0.27 0.89	< 0.001***	
			America	− 0.17	0.2	− 0.55 0.23	0.419	
	5 mm	7 (1336)	Asia (ref.)				0.004**	61.70%
			Europe	0.48	0.16	0.17 0.79	0.002**	
			America	− 0.11	0.2	− 0.50 0.28	0.582	
C	1 mm	8 (1652)	Asia (ref.)				< 0.001***	87.10%
			Europe	0.09	0.06	− 0.03 0.22	0.128	
			America	− 0.44	0.09	− 0.61 − 0.26	< 0.001***	
			Africa	− 0.38	0.09	− 0.55 − 0.2	< 0.001***	
	3 mm	6 (1006)	Asia (ref.)				0.005**	63.70%
			Europe	0.28	0.17	− 0.06 0.61	0.105	
			America	0.21	0.21	− 0.86 − 0.03	0.035*	
	5 mm	6 (840)	Asia (ref.)				0.012*	58.70%
			Europe	0.33	0.16	0.01 0.64	0.044*	
			America	− 0.28	0.2	− 0.68 0.11	0.159	

*N* study number, *SE* standard error, *CI* confidence interval, *I*^2^ I-squared, *QH* Cochran´s Q, *R*^2^ R-squared

^*^p < 0.05; **p < 0.01; ***p < 0.001 (Strength of statistical significance)

The geographical setting was only significant at the middle root in C reporting thicker FAB in studies from Africa than those from Asia (R2 = 53%) (Additional file 4). Moreover, this geographic setting seemed to influence on CEJ-FBC distance. On the other hand, the studies propensity for confounding- and selection-bias tended to overestimate CEJ-FBC distance (Additional file 5).

There was not an effect on the other modifiers (e.g. periodontal status, smoking, voxel size) on either FAB thickness or CEJ-FBC distance. Regarding the prevalence of dehiscences and fenestrations, studies with controlled periodontal status and smoking habit as an inclusion criterion tended to have lower prevalences.

### Evidence certainty

Evidence certainty has shown moderate quality in most analysis subsets. Still, it proves to be low-quality because of risk of bias and imprecision in others, as demonstrated by the SoF tables for primary—(Table 2) and secondary-outcomes (Table 3). Full assessment of certainty evidence by GRADE tool is presented in Additional file 6 (Table S1, S2 and S3).

## Discussion

This systematic review was aimed to analyze the FAB thickness of anterior maxillary teeth measured by CBCT scans. Of 29 included studies, 17 assessed the FAB using the FBC and 12 the CEJ as anatomical references to their measurements. The included studies are observational, and most showed a moderate quality of evidence. No randomized studies were detected; due to the nature of the included data, the results should be interpreted with caution.

The main findings of this meta-analysis showed that the FAB is thin, and increases in thickness when the tooth analyzed is located in a more posterior area. Different FAB values were observed depending on the anatomical reference point used. Anterior teeth (CI, LI and C) showed a mean FAB thickness ≤ 1 mm and maxillary premolars between 1–2 mm. Results from studies taking CEJ as reference point showed a thinner FAB, with a more homogenous thickness pattern, when compared with those using FBC as the anatomical reference point (Fig. 4). The geographical setting proved to be an effect modifier that could explain up to 87% of the heterogeneity in FAB thickness measures from FBC, being Asian populations which showed the lowest FAB thickness values. Patient age, gender and gingival phenotype also influenced the results, as evidenced by the subgroups analysis. Patients over 50 years of age and females exhibited a thinner FAB at maxillary incisors and canines. Noteworthy to mention, the factor with the greatest influence on FAB thickness was the gingival phenotype—with clinically thicker gingival phenotypes being associated with thicker FAB phenotypes in all anterior teeth.

Regarding secondary outcomes, CEJ-FBC distance measured between 2 and 2.5 mm in all of the analyzed teeth. Age groups over 50 years exhibited greater distances at maxillary incisors and canines, and males also showed a trend for higher CEJ-FBC distance. The prevalence of bone dehiscence at maxillary incisors and canines oscillates around 12% to 20%; while the prevalence of bone fenestration was 6.4% at CI, and oscillates between 21 to 23% in LI and C. Nevertheless, it should be stressed that findings of the present work entailed a high heterogeneity that was sought through meta-regression analyses.

Systemic factors could also influence the CEJ-FBC distance; diabetics, hypertensive, thyroid disorders, hyperlipidemia, depression and asthma has been related to greater CEJ-FBC distance [12]. Progressive periodontal attachment loss throughout life also results in an increased CEJ-FBC distance [16]. A recent study has published an inverse relationship between CEJ-FBC distance and FAB thickness at anterior maxillary teeth [54]. Even some authors have also associated shorter CEJ-FBC distances with thicker gingival biotype [44, 45].

The scientific literature has described that FAB thickness can either be evaluated intraoperatively 1 mm below the alveolar bone crest after tooth extraction [11] or measured by CBCT at different apico-coronal levels before or after exodontia [16, 24, 38]. Though CBCT may optimize periodontal and implant treatment planning [55–57]; the employment of 3D technologies to obtain bidimensional measures has been the subject of intense debate in the literature provided by ex-vivo and in-vitro studies [36]. It is because some factors like patient motion reduce accuracy and reliability of linear measurements on CBCT images, device-specific exposure parameters, manual versus automated procedures, metallic artifacts of dental implant and bioceramic materials [36, 58, 59]. However, no significant differences were reported for bone thickness values (− 0.07 mm) between CBCT and direct measurements with calipers in vivo [60]. Besides, CEJ anatomical reference registration is usually difficult, though its impact on bone height measurements is to be around 0.01 mm, this is a lower difference than obtained with cusp tip (0.1 mm) as landmark [60]. These values are lower than the maximum errors reported for CBCT-derived tooth surfaces measures in vivo (± 0.2 mm) [61]. Also, this measurement variability at tooth surfaces level with CBCT, in vivo, is not that different from those observed for FAB thickness at CI, LI and C. In the present work, FAB measurement standard deviations in CI, LI, and C were around (± 0.2 to ± 0.29 mm) when measured from FBC; and around (± 0.07 to ± 0.19 mm) when measured from the CEJ.

The voxel size is related to image resolution; values of 0.3 to 0.4 mm are considered suitable for planification in oral implantology [57]. The smaller the voxel size, the greater the noise, but the higher the spatial resolution [62]. The isotropic voxel size reported among the included studies was around 0.15–0.4 mm. This covariate did not show a significant effect across the parameters assessed.

To ensure appropriate post-extraction treatment, an analysis of the FAB dimensions through CBCT scanning in the region of the tooth to be extracted can offer valuable information regarding the bone volume and the morphology of the future implant site [19, 43]. In humans, bone modeling in single extraction sites is mainly localized to the central aspect of the FAB, whereas proximal aspects are well maintained by the periodontal ligaments of the adjacent teeth [9]. FAB height influences the position of the mucosal margin around teeth and implants, whereas FAB thickness exerts an influence upon the facial convexity of the alveolar process at the emerging implant crown [20]. Classically, it has been suggested that a minimum FAB thickness of 2 mm is an important feature for the maintenance of vertical crestal resorption following tooth extraction [63–65]—this feature rarely being found in anterior maxillary teeth. Recent studies have demonstrated that 1 mm of FAB could be enough to minimize alveolus postextraction morphological changes [6, 7]. Due to the different thickness along the apico-coronal dimension of the FAB in a single tooth, postextraction morphological changes are not expected to occur in the same manner along the entire alveolus. The present review observed an increase in FAB thickness as the tooth to be analyzed is located in a more posterior area, so morphological changes after exodontia of anterior maxillary teeth with < 1 mm FAB thickness are expected to be more pronounced than those occurred at maxillary premolars with 1–2 mm FAB thickness.

A well-documented technique for minimizing postextraction morphological changes is alveolar ridge preservation, though FAB has usually resorbed unavoidably [66]. Several meta-analyses and systematic reviews have shown some benefits of alveolar ridge preservation in maintaining ridge dimensions when compared to spontaneous socket healing [67, 68]. A recent meta-analysis could not determine a superior alveolar ridge preservation approach. However, xenogenic or allogenic materials covered with an absorbable collagen membrane were associated with the most favorable outcomes in maintaining horizontal dimensions [69]. Although benefits were observed with alveolar ridge preservation in terms of a decrease in morphological changes after tooth extraction, some degree of horizontal and vertical bone loss should still be expected [70]. Baseline FAB thickness influences the postextraction morphological changes after alveolar ridge preservation; sites exhibiting > 1 mm of FAB thickness underwent less vertical and horizontal bone resorption than sites presenting < 1 mm of FAB. The horizontal bone reduction values were 1.29 ± 0.2 mm in sites with thick FAB and 3.22 ± 0.2 mm in sites with thin FAB [71]. Concerning an immediate implant approach, a careful analysis of FAB integrity and thickness is mandatory [10, 72]. A recent prospective cohort study [73] of immediate implants with immediate provisionalization observed, at one year of follow-up, that bone resorption and peri-implant soft tissue recession were massive when the preoperative FAB thickness was < 0.5 mm compared to 0.5–1.5 mm.

Despite the efforts like the comprehensive electronic- and complementary-literature search carried out, and the critical qualitative and quantitative synthesis of meta-evidence; the high statistical heterogeneity observed is a consequence of both clinical (e.g. sex, age, geographical setting diversity) and methodological (e.g. propensity for confounding and selection bias) heterogeneity sources among included studies due to its observational nature. Though a great extent of the observed heterogeneity was explained through meta-regression analyses, it is important to emphasize that results should be interpreted with caution because of the limitations mentioned above. The aspects mentioned above were integrated in a transparent manner using SoF tables using the GRADE approach, and using a threshold to address imprecision of the results as complementary basis. This approach allows us to reach an overall certainty of meta-analytical data, making evidence more useful, shedding light limitations that must be improved in future studies.

Some recommendations arose after this comprehensive analysis of meta-evidence: future prospective studies (comparative and randomized) should conduct according to power calculations based on the anatomical references (CEJ or FBC) and apical-coronal cut-off points. Also, it is highly recommendable to take into account the gingival phenotype, periodontal status and smoking habits feature during selection or in statistical analysis. A tentative clinical setting is the immediate implants in the anterior maxilla, allowing both preoperative CBCT imaging measurement of FAB, and in-situ before implant insertion, or using non-ionizing imaging techniques like magnetic resonance. The relevancy of this topic is not only restricted to surgical procedures, but also in orthodontics and restorative dentistry.

## Conclusions

Within the limitations of the present systematic review, it can be concluded that:

- Facial alveolar bone (FAB) becomes thicker as the tooth is located in a more posterior area. FAB is thinner in the first coronal and middle root locations, and thicker in-between. Incisors and canines presented a mean FAB thickness < 1 mm at all measurement locations. Premolars showed a mean FAB thickness between 1–2 mm over the whole apico-coronal dimension.
- With respect to the modifying factors in FAB thickness, females and age groups over 50 years showed significant thinner FAB at some locations of incisors and canines. However, the factor with the greatest influence on FAB at all dimensions assessed was gingival phenotype. The geographical setting proved to be an effect modifier that could explain heterogeneity in FAB thickness. Asian populations showed thinner FAB when compared with those from Europe in incisors and canines.
- The CEJ-FBC distance is quite constant at all maxillary teeth analyzed, and proved greater in individuals over 50 years of age at the incisors and canines.

## Supplementary Information

**Additional file 1.** Studies excluded by full-text assessment.

**Additional file 2.** Characteristics and major inferences of the selected studies.

**Additional file 3.** Influence of age and sex subgroups on FAB thickness at different points from FBC and influence of the gingival phenotype on FAB thickness at different points.

**Additional file 4.** Measurement of FAB thickness with CEJ as reference point analyzed by meta-regression of the geographical study setting at CI, LI and C.

**Additional file 5.** Measurement of CEJ-FBC distance analyzed by meta-regression of study geographical setting CI, LI and C.

**Additional file 6.** Supplementary Table S1. Assessment of the certainty of evidence by GRADE tool for FAB thickness considering FBC as anatomical reference. Supplementary Table S2. Assessment of the certainty of evidence by GRADE tool for FAB thickness considering CEJ as anatomical reference. Supplementary Table S3. Assessment of the certainty of evidence by GRADE tool for CEJ-FBC distance.

## Data Availability

All data generated or analyzed during this study are included in this published article and the supplementary files for transparency.

## Abbreviations

CBCT: Cone-beam computed tomography
FAB: Facial alveolar bone
CEJ: Cementoenamel junction
CI: Central incisor
LI: Lateral incisor
C: Canine
1PM: First premolar
2PM: Second premolar
