# Supplementary material for: Facial alveolar bone thickness and modifying factors of anterior maxillary teeth: a systematic review and meta-analysis of cone-beam computed tomography studies
Source: BMC Oral Health. 2021 Mar 22;21:143. doi: 10.1186/s12903-021-01495-2 (PMC7986564; doi:10.1186/s12903-021-01495-2)
Supplement: Supplementary file 1 — Additional file 1. Studies excluded by full-text assessment. [file 12903_2021_1495_MOESM1_ESM.docx]

**Additional file 1.** Studies excluded after full-text assessment.

| **N** | **Referecences screened by full-text assessment** | **Reason for exclusion by full-text** |
| --- | --- | --- |
| 1 | Adiguzel Ozkan, Belgin Ceren Aktuna, Falakaloglu Seda, Cangul Suzan, Akkus Zeki. Maxillary cortical bone thickness in a south- eastern anatolian population: A cone-beam computed tomography study. Med Sci Monit 2017;23:5812–7. Doi: 10.12659/MSM.906229. | Non reproducible way of measuring |
| 2 | Ahn Hyo Won, Moon Sung Chul, Baek Seung Hak. Morphometric evaluation of changes in the alveolar bone and roots of the maxillary anterior teeth before and after en masse retraction using cone-beam computed tomography. Angle Orthod 2013;83(2):212–21. Doi: 10.2319/041812-325.1. | Patients with premolars extraction and orthodontic treatment |
| 3 | Al-Jaf Nagham M., Abdul Wahab Rohaya Megat, Abu Hassan Mohamed Ibrahim. Buccal cortical bone thickness in different sagittal skeletal relationship. Orthod Waves 2018;77(4):220–5. Doi: 10.1016/j.odw.2018.08.001. | Analyzing interradicular bone, not facial alveolar bone at maxillary teeth |
| 4 | Arief Erry Mochamad, Ngee Teh Thian, Hassan Akram, Shaari Ramizu, Alam Mohammad Khursheed, Daud Firdaus. Cone beam computed tomographic (CBCT) evaluation of maxillary anterior alveolar bone. Int Med J 2013;20(3):326–8. | Sample size (26 patients) |
| 5 | Bonta Hernán, Carranza Nelson, Gualtieri Ariel F, Rojas Mariana A. Morphological characteristics of the facial bone wall related to the tooth position in the alveolar crest in the maxillary anterior. Acta Odontol Latinoam 2017;30(2):49–56. | Non reproducible way of measuring |
| 6 | Chen Sheng-Hong, Chan Hsun-Liang, Lu Yongning, et al. A Semi-automatic Algorithm for Preliminary Assessment of Labial Gingiva and Alveolar Bone Thickness of Maxillary Anterior Teeth. Int J Oral Maxillofac Implants 2017;32(6):1273–80. Doi: 10.11607/jomi.5566. | Sample size (11 patients) |
| 7 | Coşkun İpek, Kaya Burçak. Relationship between alveolar bone thickness, tooth root morphology, and sagittal skeletal pattern: A cone beam computed tomography study. J Orofac Orthop 2019;80(3):144–58. Doi: 10.1007/s00056-019-00175-9. | Analyzing interradicular bone, not facial alveolar bone at maxillary teeth |
| 8 | Digregorio Michele Vito, Fastuca Rosamaria, Zecca Piero Antonio, Caprioglio Alberto, Lagravère Manuel O. Buccal bone plate thickness after rapid maxillary expansion in mixed and permanent dentitions. Am J Orthod Dentofac Orthop 2019;155(2):198–206. Doi: 10.1016/j.ajodo.2018.03.020. | Patients with deciduous teeth |
| 9 | Dong Zheng Jie, Xu Kan. Study of angle between maxillary incisor and alveolar bone using cone-beam CT. Shanghai Kou Qiang Yi Xue 2017;26(4):458–60. | Not analyzing facial alveolar bone thickness |
| 10 | Esfahanizadeh Nasrin, Daneshparvar Niloufar, Askarpour Farinaz, Akhoundi Nasrin, Panjnoush Mehrdad. Correlation Between Bone and Soft Tissue Thickness in Maxillary Anterior Teeth. J Dent (Tehran) 2016;13(5):302–8. | Patients with absences at the anterior maxillary teeth |
| 11 | Frost Natalie A., Mealey Brian L., Jones Archie A., Huynh-Ba Guy. Periodontal Biotype: Gingival Thickness as It Relates to Probe Visibility and Buccal Plate Thickness. J Periodontol 2015;86(10):1141–9. Doi: 10.1902/jop.2015.140394. | Patients with absences at the anterior maxillary teeth |
| 12 | Frumkin Nathalie, Via Shlomo, Klinger Avigdor. Evaluation of the width of the alveolar bone in subjects with different gingival biotypes: A prospective cohort study using cone beam computed tomography. Quintessence Int (Berl) 2017;48(3):209–16. Doi: 10.3290/j.qi.a37642. | Sample size (16 patients) |
| 13 | Fuentes Ramón, Flores Tania, Navarro Pablo, Salamanca Carlos, Beltrán Víctor, Borie Eduardo. Assessment of buccal bone thickness of aesthetic maxillary region: A cone-beam computed tomography study. J Periodontal Implant Sci 2015;45(5):162–8. Doi: 10.5051/jpis.2015.45.5.162. | Non reproducible way of measuring |
| 14 | Germec-Cakan Derya, Tozlu Murat, Ozdemir Fulya. Cortical bone thickness of the adult alveolar process--a retrospective CBCT study. Aust Orthod J 2014;30(1):54–60. | Analyzing ridge thickness, not facial alveolar bone at maxillary teeth |
| 15 | Giglou K., Batal H., Mehra P. Does Sagittal Root Position Affect Buccal or Palatal Bone Thickness in the Anterior Esthetic Zone? J Oral Maxillofac Surg 2017;75(10):e354–5. Doi: 10.1016/j.joms.2017.07.049. | Non reproducible way of measuring |
| 16 | Gonen Zeynep Burcin, Alkan Alper, Ekizer Abdullah, Kutuk Nukhet, Tasdemir Zekeriya. Evaluation of Vestibular Bone Thickness in Class I Malocclusion Treatment With Corticotomy-Assisted Rapid Orthodontics. J Craniofac Surg 2019;30(8):e727–33. Doi: 10.1097/SCS.0000000000005736. | Orthodontic treatment with corticotomies |
| 17 | Jin G. C., Kim K. D., Roh B. D., Lee C. Y., Lee S. J. Buccal bone plate thickness of the Asian people. J Endod 2005;31(6):430–4. Doi: 10.1097/01.don.0000145430.35906.fa. | Non reproducible way of measuring |
| 18 | Jin Seong Ho, Park Jun Beom, Kim Namryang, et al. The thickness of alveolar bone at the maxillary canine and premolar teeth in normal occlusion. J Periodontal Implant Sci 2012;42(5):173–8. Doi: 10.5051/jpis.2012.42.5.173. | Sample size (20 patients) |
| 19 | Kim Yun Jeong, Park Ji Man, Kim Sungtae, et al. New method of assessing the relationship between buccal bone thickness and gingival thickness. J Periodontal Implant Sci 2016;46(6):372–81. Doi: 10.5051/jpis.2016.46.6.372. | Sample size (20 patients) |
| 20 | La Rocca Andres Pascual, Alemany Antonio Santos, Levi Paul, Juan Monica Vicario, Molina Jose Nart, Weisgold Arnold S. Anterior maxillary and mandibular biotype: Relationship between gingival thickness and width with respect to underlying bone thickness. Implant Dent 2012;21(6):507–15. Doi: 10.1097/ID.0b013e318271d487. | Sample size (15 patients) |
| 21 | Lee Seung Lok, Kim Hee Jung, Son Mee Kyoung, Chung Chae Heon. Anthropometric analysis of maxillary anterior buccal bone of Korean adults using cone-beam CT. J Adv Prosthodont 2010;2(3):92–6. Doi: 10.4047/jap.2010.2.3.92. | Sample size (20 patients) |
| 22 | Ozdemir Fulya, Tozlu Murat, Germec-Cakan Derya. Cortical bone thickness of the alveolar process measured with cone-beam computed tomography in patients with different facial types. Am J Orthod Dentofac Orthop 2013;143(2):190–6. Doi: 10.1016/j.ajodo.2012.09.013. | Analyzing ridge thickness, not facial alveolar bone at maxillary teeth |
| 23 | Pascual Andrés, Barallat Lucía, Santos Antonio, et al. Comparison of Periodontal Biotypes Between Maxillary and Mandibular Anterior Teeth: A Clinical and Radiographic Study. Int J Periodontics Restorative Dent 2017;37(4):533–9. Doi: 10.11607/prd.2848. | Sample size (15 patients) |
| 24 | Ramanauskaite Ausra, Becker K., Kassira H. C., Becker J., Sader R., Schwarz F. The dimensions of the facial alveolar bone at tooth sites with local pathologies: a retrospective cone-beam CT analysis. Clin Oral Investig 2019:10.1007/s00784-019-03057–x. Doi: 10.1007/s00784-019-03057-x. | Teeth included with endodontic treatment, periapical infection, and local pathologies |
| 25 | Rojo-Sanchis Julio, Peñarrocha-Oltra David, Peñarrocha-Diago Miguel, Zaragozí-Alonso Regino. Relation between the distance from the cementoenamel junction to the bone crest and the thickness of the facial bone in anterior maxillary teeth: A cross-sectional tomographic study. Med Oral Patol Oral y Cir Bucal 2019;24(3):e409–15. Doi: 10.4317/medoral.22802. | Teeth included with different periodontal status |
| 26 | Sadek Mais M., Sabet Noha E., Hassan Islam T. Alveolar bone mapping in subjects with different vertical facial dimensions. Eur J Orthod 2014;37(2):194–201. Doi: 10.1093/ejo/cju034. | Analyzing ridge thickness, not facial alveolar bone at maxillary teeth |
| 27 | Sendyk Michelle, de Paiva João Batista, Abrão Jorge, Rino Neto José. Correlation between buccolingual tooth inclination and alveolar bone thickness in subjects with Class III dentofacial deformities. Am J Orthod Dentofac Orthop 2017;152(1):66–79. Doi: 10.1016/j.ajodo.2016.12.014. | Non reproducible way of measuring |
| 28 | Sendyk Michelle, Linhares Daniele Sigal, Pannuti Claudio Mendes, de Paiva João Batista, Neto José Rino. Effect of orthodontic treatment on alveolar bone thickness in adults: A systematic review. Dental Press J Orthod 2019;24(4):34–45. Doi: 10.1590/2177-6709.24.4.034-045.oar. | Patients with orthodontic treatment |
| 29 | Shen Jian wei, He Fu ming, Jiang Qiao hong, Shan Hai qin. [Measurement of facial bone wall thickness of maxillary anterior teeth and premolars on cone beam computed tomography images]. Zhejiang Da Xue Xue Bao Yi Xue Ban 2012;41(3):234–8. | Non reproducible way of measuring |
| 30 | Vera Carolina, De Kok Ingeborg J, Reinhold Dominik, et al. Evaluation of buccal alveolar bone dimension of maxillary anterior and premolar teeth: a cone beam computed tomography investigation. Int J Oral Maxillofac Implants 2012;27(6):1514–9. | Patients with abscences and dental implants in the anterior maxillae |
| 31 | Yang Gang, Hu Wen jie, Cao Jie, Liu Deng gao. [Measurement of sagittal root position and the thickness of the facial and palatal alveolar bone of maxillary anterior teeth]. Zhonghua Kou Qiang Yi Xue Za Zhi 2013;48(12):716–20. | Sample size (23 patients) |
| 32 | Yodthong Nuengrutai, Charoemratrote Chairat, Leethanakul Chidchanok. Factors related to alveolar bone thickness during upper incisor retraction. Angle Orthod 2013;83(3):394–401. Doi: 10.2319/062912-534.1. | Patients with orthodontic treatment |
| 33 | Younes Faris, Eghbali Aryan, Raes Margot, De Bruyckere Thomas, Cosyn Jan, De Bruyn Hugo. Relationship between buccal bone and gingival thickness revisited using non-invasive registration methods. Clin Oral Implants Res 2016;27(5):523–8. Doi: 10.1111/clr.12618. | Sample size (21 patients) |
| 34 | Zhang Wenjian, Skrypczak Adam, Weltman Robin. Anterior maxilla alveolar ridge dimension and morphology measurement by cone beam computerized tomography (CBCT) for immediate implant treatment planning. BMC Oral Health 2015;15(1):65. Doi: 10.1186/s12903-015-0055-1. | Analyzing ridge thickness, not facial alveolar bone at maxillary teeth |
