# Supplementary material for: Facial alveolar bone thickness and modifying factors of anterior maxillary teeth: a systematic review and meta-analysis of cone-beam computed tomography studies
Source: BMC Oral Health. 2021 Mar 22;21:143. doi: 10.1186/s12903-021-01495-2 (PMC7986564; doi:10.1186/s12903-021-01495-2)
Supplement: Supplementary file 2 — Additional file 2. Characteristics and major inferences of the selected studies. [file 12903_2021_1495_MOESM2_ESM.docx]

| **Author/year** | **Journal** | **Design** | **Sample size (gender)** | **Mean age (range)** | **CBCT settings** | **Location measurement** | **Teeth included** | **FAB Thickness** |
| --- | --- | --- | --- | --- | --- | --- | --- | --- |
| Amid/2017 | *Arch. Oral Biol* | Cross-sectional (CS) | 144 (60 men, 84 women) | 44.62 (NR) | Soredex: FOV (12 x 8) and 200 mm voxel size | 2, 4 and 6 mm from CEJ | 621 (CI, LI, C) | **CI**: 2 mm (0.43±0.46), 4 mm (0.85±0.32), 6 mm (0.82±0.3) |
|  |  |  |  |  |  |  |  | **LI**: 2 mm (0.38±0.43), 4 mm (0.9±1.7), 6 mm (0.89±0.38) |
|  |  |  |  |  |  |  |  | **C**: 2 mm (0.46±0.57), 4 mm (0.92±0.45), 6 mm (0.9±0.43) |
| Braut/2011 | *Int. J. Periodontics Restorative Dent* | Retrospective cohort study (R-CHT) | 125 (60 men, 65 women) | 47.3 (17-84) | 3D Accuitomo: FOV (4X4 or 6x6 or 8x8). Monitor Eizo Nanao | 4 mm and middle root apical to CEJ | 498 (CI, LI, C, 1PM) | **CI**: P1 (0.47±0.5), P2 (0.59±0.57) |
|  |  |  |  |  |  |  |  | **LI**: P1 (0.54±0.56), P2 (0.55±0.51) |
|  |  |  |  |  |  |  |  | **C**: P1 (0.45±0.52), P2 (0.57±0.52) |
|  |  |  |  |  |  |  |  | **1PM**: P1 (0.73±0.47), P2 (0.65±0.49) |
| Cook/2011 | *Int. J. Periodontics Restorative Dent* | Case-control study | 60 (NR) | NR | Accuitomo: FOV: 2,5; 1 mm slice | 4, 6, 8 and 10 mm from CEJ | 360 (CI, LI, C) | **CI**: 4 mm (0.59±0.11), 6 mm (0.66±0.12), 8 mm (0.77±0.14), 10 mm (0.9±0.23) |
|  |  |  |  |  |  |  |  | **LI**: 4 mm (0.58±0.14), 6mm (0.64±0.13), 8mm (0.74±0.15), 10mm (0.87±0.26) |
|  |  |  |  |  |  |  |  | **C**: 4mm (0.47±0.14), 6mm (0.54±0.12), 8 mm (0.63±0.14), 10 mm (0.73±0.22) |
| Demircan/2015 | *Implant Dent* | CS | 66 (37 men, 28 women) | 38.7 (18-84) | Galileos: FOV 15 x 12 cm, 1 mm slice, voxel size 70 mSv | 1, 2 and 5 mm from FBC | 240 (CI, LI) | **CI**: 1 mm (0.72±0.17), 2 mm (0.85±0.19), 5 mm (0.75±0.17) |
|  |  |  |  |  |  |  |  | **LI**: 1 mm (0.76±0.22), 2 mm (0.83±0.24), 5 mm (0.7±0.22) |
| D’Silva 2019 | *J. Periodontol* | CS | 73 (42 female, 31 male) | 58 ± 14.7 (22-84) | i-CAT: 1 mm slice | 4 mm and middle root apical to CEJ | 334 (CI, LI, C) | **CI**: P1 0.89±0.42), P2 (0.86±0.37) |
|  |  |  |  |  |  |  |  | **LI:** P1 (0.96±0.5), P2 (0.88±0.64) |
|  |  |  |  |  |  |  |  | **C:** P1 (0.9±0.61), P2 (0.65±0.5) |
| El Nahass/2015 | *Clin. Oral Implants Res* | CS | 73 (42 female, 31 male) | 42.3 (24-56) | Cranex 3D: FOV 61 x 41, 1mm slice | 1, 2 and 4 mm from FBC | 146 (CI, LI) | **CI**: 1 mm (0.72±0.19), 2 mm (0.78±0.18), 4 mm (0.81±0.1) |
| Farahamnd/2017 | *Int. J. Periodontics Restorative Dent* | CS | 132 (67 male, 65 female) | NR (18-80) | 1 mm slice | 2, 5 and 8 from FBC | 792 (CI, LI, C) | **CI:** 1 mm (0.73±0.19), 2 mm: (0.84±0.23), 4 mm (0.84±0.25) |
|  |  |  |  |  |  |  |  | **LI:** 2 mm (0.75±0.62), 5 mm (0.69±0.64), 8 mm (0.47±0.53) |
|  |  |  |  |  |  |  |  | **C:** 2 mm (0.79±0.69), 5 mm (0.71±0.64), 8 mm (0.52±0.57) |
| Gakonyo/2018 | *Int. J. Oral Maxillofac. Implants* | CS | 184 (85 men, 99 female) | 39.28 (18-81) | Galileos: FOV 15, voxel size 0.3, 85 Kv and 7 mA | 4 mm and middle root apical to CEJ | 1104 (CI, LI, C) | **CI**: P1 (0.58±38), P2 (0.68±0.3) |
|  |  |  |  |  |  |  |  | **LI**: P1 (0.56±0.4), P2 (0.59±0.36) |
|  |  |  |  |  |  |  |  | **C**: P1 (0.5±0.46), P2 (0.52±0.41) |
| Ganji/2017 | *J Esthet Restor Dent* | CS | 32 (NR) | 20-35 | CranexTM 3Dx: FOV 100x995 mm, voxel size 0.2, 96 Kv and 11 mA, 12 seconds | 4 mm and middle root apical to CEJ | 128 (1PM, 2PM) | **1PM**: (1.17±0.34) |
|  |  |  |  |  |  |  |  | **2PM**: (1.34±0.6) |
| Ghassemian/2012 | J. Periodontol | CS | 66 (31 males, 36 female) | 39.9 (17-69) | Lightspeed: 1 mm slice, FOV: 25, 120 kV, 100 mA | 1, 2, 3, 4 and 5 mm apical to FBC | 396 (IC, IL, C) | **CI**: 1mm (1.13±0.36), 2mm (1.41±0.41), 3 mm (1.43±0.44), 4 mm (1.37±0.45), 5 mm (1.22±0.53) |
|  |  |  |  |  |  |  |  | **LI**: 1mm (1.21±0.34), 2mm (1.56±0.46), 3 mm (1.66±1.53), 4 mm (1.57±0.61), 5 mm (1.32±0.57) |
|  |  |  |  |  |  |  |  | **C**: 1 mm (1.13±0.37), 2 mm (1.42±0.4), 3 mm (1.53±0.47), 4 mm (1.43±0.52), 5 mm (1.31±0.57) |
| Gluckman/2018 | *J Prosthet Dent* | CS | 150 (67 males, 83 females) | 49.4 (18-89) | Carestream 9300 3D: FOV: 5×5, 8×8, 10×5, 10×10, 17×6, 17×11, 17×13.5 | 1 mm from the FBC, at the apex and midway between 1 mm from the FBC and the apex | 591 (CI, LI, C) | **CI:** P1 (0.6±0.3), P2 (0.5±0.3), P3 (1.2±0.8) |
|  |  |  |  |  |  |  |  | **LI:** P1 (0.7±0.3), P2 (0.5±0.4), P3 (1.5±1.2) |
|  |  |  |  |  |  |  |  | **C:** P1 (0.6±0.3), P2 (0.5±0.3), P3 (1.4±1) |
| Januário/2011 | *Clin. Oral Implants Res* | CS | 250 (118 males, 132 female) | 37.9 (17-66) | i-CAT: FOV: 6 x 17, voxel 0.2, 40 seconds | 1, 3 and 5 from FBC | 1500 (CI, LI, C) | **CI**: 1 mm (0.6±0.3), 3 mm (0.6±0.4), 5 mm (0.5±0.3) |
|  |  |  |  |  |  |  |  | **LI**: 1mm (0.7±0.3), 3 mm (0.7±0.4), 5 mm (0.5±0.4) |
|  |  |  |  |  |  |  |  | **C**: 1 mm (0.6±0.3), 3 mm (0.6±0.4), 5 mm (0.6±0.4) |

**Additional file 2.** Characteristics and major inferences of the selected studies.

**Additional file 2** (continued).

| **Author/year** | **Journal** | **Design** | **Sample size (gender)** | **Mean age (range)** | **CBCT settings** | **Location measurement** | **Teeth included** | **FAB Thickness** |
| --- | --- | --- | --- | --- | --- | --- | --- | --- |
| Jung/2017 | *Imaging Sci Dent* | CS | 199 (100 males, 99 females) | 28.3 (20-50) | PaX-Zenith 3D: FOV: 16×14or 12×9, voxel size 0.2, 5.7 mA, 110 kV, 24 seconds | Bone crest, 2, 4 and 6 mm from the FBC and at the apex | 796 (CI, LI) | **CI:** bone crest (0.79±0.16), 2mm (0.91±0.24), 4 mm (0.86±0.27), 6 mm (0.87±0.35), apex (1.18±0.53) |
|  |  |  |  |  |  |  |  | **LI:** bone crest (0.70±0.26), 2mm (0.67±0.42), 4 mm (0.40±0.38), 6 mm (0.26±0.31), ápex (0.81±0.63) |
| Kheur/2015 | *Implant Dent* | R-CHT | 150 (NR) | NR | i-CAT: 120 Kv, 18.66 mA, voxel size 0.25 | 3 mm apical CEJ, middle root, apical root | 300 (CI) | **CI**: P1 (0.92±0.38), P2 (0.89±0.52), P3 (1.57±0.88) |
| Khoury/2016 | *Implant Dent* | CS | 47 (16 males, 31 females) | 34 (21-48) | NewTom: 1 mm slice | 4, 6, 8 and 10 from CEJ | 282 (CI, LI, C) | **CI:** 4 mm (1.07±0.38), 6 mm (1.01±0.35), 8 mm (0.93±0.32), 10 mm (0.95±0.41) |
|  |  |  |  |  |  |  |  | **LI:** 4 mm (1.1±0.56), 6 mm (0.93±0.65), 8 mm (0.66±0.64), 10 mm (0.57±0.61) |
|  |  |  |  |  |  |  |  | **C:** 4 mm (0.94±0.55), 6 mm (0.81±0.6), 8 mm (0.63±0.56), 10 mm (0.47±0.46) |
| Koç/2019 | *Cumhur Dent J* | CS | 62 (29 males, 33 females) | 19-60 | KaVo 3D eXam | At the crest, 1 and 2 mm apical FBC | 186 (CI, LI, C) | **CI:** bone crest (0.69±0.16), 1 mm (0.74±0.18), 2 mm (0.76±0.21) |
|  |  |  |  |  |  |  |  | **LI:** bone crest (0.74±0.19), 1 mm (0.81±0.23), 2 mm (0.85±0.29) |
|  |  |  |  |  |  |  |  | **C:** bone crest (0.97±0.26), 1 mm (1.04±0.3), 2 mm (1.06±0.35) |
| López-Jarana/2018 | *BMC Oral Health* | CS | 49 (19 males, 30 females) | 40.3 | Planmeca ProMax 3D: FOV 200 mm, voxel 200 mSV, 90 kV, 10 mA, 1 mm slice | 1 and 4 mm apical FBC and at the apex | 208 (CI, LI, C, 1PM, 2PM) | **CI:** 1 mm (1.07±0.49), 4 mm (1.02±0.49), apex (1.6±0.95) |
|  |  |  |  |  |  |  |  | **LI:** 1 mm (0.99±0.44), 4 mm (1.02±0.49), apex (1.6±0.95) |
|  |  |  |  |  |  |  |  | **C:** 1 mm (1.04±0.39), 4 mm (1.27±1.95), apex (1.26±0.68) |
|  |  |  |  |  |  |  |  | **1PM:** 1 mm (1.2±0.67), 4 mm (1.43±0.95), apex (2.19±1.68) |
|  |  |  |  |  |  |  |  | **2PM:** 1 mm (1.2±0.67), 4 mm (1.43±0.95), apex (2.19±1.68) |
| Morad/2014 | *Craniofac. Surg* | CS | 152 (NR) | 44.4 (16-79) | NewTom: 110Kv, 10-20 Ma, exposure 3.6 seconds | 1,2,3,4,5 mm apical FBC | 276 (CI, LI, C) | **CI**: 1 mm (1.04±0.17), 2 mm (1.14±0.2), 3 mm (1.13±0.22), 4 mm (1.07±0.23), 5 mm (1.02±0.25) |
|  |  |  |  |  |  |  |  | **LI**: 1 mm (1.11±0.17), 2 mm (1.26±0.19), 3 mm (1.24±0.24), 4 mm (1.08±0.25), 5 mm (0.91±0.25) |
|  |  |  |  |  |  |  |  | **C**: 1 mm (1.04±0.22), 2 mm (1.14±0.24), 3 mm (1.1±0.22), 4 mm (1.01±0.19), 5 mm (0.9±0.2) |
| Nowzari/2010 | *Clin Implant Dent Relat Res* | Retrospective | 101 (53 men, 48 women) | 48.5 (15-82) | NewTom 3G: 110Kv, 1-15mA, 60Sv, 12FOV; Galileos: 85Kv, 5-7mA, 15FOV, 68Sv | 1,2,3,4,5,6,7,8,9,10 mm apical FBC | 202 (CI) | **CI**: 1 mm (1±0.3); 2 mm (1.15±0.35), 3 mm (1.2±0.4); 4 mm (1.1±0.4); 5 mm (1.1±0.4); 6 mm (1.05±0.45); 7 mm (1.15±0.55); 8 mm (1.05±0.7); 9 mm (1.1±0.8); 10 mm (1.25±1.05) |
| Rojo-Sanchis/2017 | *J. Oral Implantol* | CS | 44 (25 men, 19 women) | 42.7 (18-60) | Planmeca Promax 3D: voxel 150 mSV, 90 Kv, 10 mA, FOV (4x4) | 1, 2, 3 and 5 mm from FBC | 144 (1PM, 2PM) | **1PM**: 1mm (1.41±0.5), 2mm (1.68±0.72), 3mm (1.71±0.89), 5mm (1.44±1) |
|  |  |  |  |  |  |  |  | **2PM**: 1mm (1.72±0.56), 2mm (2.23±0.66), 3mm (2.43±0.82), 5mm (2.31±1.06) |
| Sheerah/2019 | *Saudi Dent J* | CS | 490 (109 men, 77 women) | 34.65 ± 11.57 (18-65) | NR | Bone crest, middle root and apex | 116 (CI, LI, C) | **CI**: bone crest (1.21±0.35), middle root (0.96±0.35), apex (1.51±0.53) |
|  |  |  |  |  |  |  |  | **LI**: bone crest (0.93±0.47), middle root (0.92±0.39), apex (1.58±0.62) |
|  |  |  |  |  |  |  |  | **C:** bone crest (0.88±0.33), middle root (0.84±0.31), apex (1.38±0.52) |
| Temple/2016 | *Clin. Oral Implants Res* | CS | 265 (119 males, 146 females) | 55.9 ± 13.7 (20-85) | Accuitomo: FOV: 4, 6, 8; 0.1 mm slice | 1, 3, 5 mm FBC | 172 (1PM, 2PM) | **1PM**: 1 mm (0.88), 3 mm (0.71), 5 mm (0.61) |
|  |  |  |  |  |  |  |  | **2PM**: 1 mm (1.32), 3 mm (1.23), 5 mm (1) |
| Uner/2019 | *Niger J Clin Pract* | CS | 160 (80 males, 80 females) | 36.86 ± 7.78 (21-53) | i-CAT: voxel size 0.3, 120 kV, 5 mA, 8-9 seconds of exposure | 3, 6 and 9 mm apical CEJ | 320 (CI) | **CI:** 3 mm (1.15±0.34), 6 mm (1.11±0.44), 9 mm (1.06±0.51) |

**Additional file 2** (continued).

| **Author/year** | **Journal** | **Design** | **Sample size (gender)** | **Mean age (range)** | **CBCT settings** | **Location measurement** | **Teeth included** | **FAB Thickness** |
| --- | --- | --- | --- | --- | --- | --- | --- | --- |
| Wang/2014 | *Int. J. Oral Maxillofac. Implants* | CS | 300 (133 men, 167 women) | 36.9 (18-60) | NewTom 3G, NNT software, 0.3mm slice thickness | 4 mm and middle root apical to CEJ | 1500 (CI, LI, C, 1PM, 2PM) | **CI:** P1 (0.8±0.4), P2 (0.8±0.3) |
|  |  |  |  |  |  |  |  | **LI**: P1 (0.7±0.4), P2 (0.7±0.3) |
|  |  |  |  |  |  |  |  | **C**: P1 (0.7±0.5), P2 (0.7±0.4) |
|  |  |  |  |  |  |  |  | **1PM**: P1 (1.2±0.6), P2 (1.2±0.6) |
|  |  |  |  |  |  |  |  | **2PM**: P1 (1.7±0.7), P2 (1.8±0.8) |
| Yuan/2019 | *West China J Stomatol* | CS | 40 (16 males and 24 females) | 26.30 ± 2.29 (23-34) | NewTom: 1 mm slice | 1, 3 and 5 mm apical FBC | 120 (CI, LI, C) | **CI:** 1 mm (0.89 ± 0.3), 3 mm (0.81 ± 0.3), 5 mm (0.67 ± 0.28) |
|  |  |  |  |  |  |  |  | **LI:** 1 mm (0.80 ± 0.33), 3 mm (0.85 ± 0.46), 5 mm (0.43 ± 0.34) |
|  |  |  |  |  |  |  |  | **C:** 1 mm (0.96 ± 0.34), 3 mm (0.96 ± 0.44), 5 mm (0.77 ± 0.37) |
| Zekry/2014 | *Clin. Oral Implants Res* | CS | 200 (74 Men, 126 Women) | 37.2 (17-82) | i-CAT; voxel 0.4; FOV (15 x 12); 120 Kv; 18-48mA | 1,3 and 5 mm apical FBC | 1303 (CI, LI, C, 1PM, 2PM) | **CI**: 1mm (0.9±0.28), 3 mm (0.89±0.3), 5 mm (0.81±0.3) |
|  |  |  |  |  |  |  |  | **LI**: 1mm (0.94±0.34), 3 mm (0.88±0.36), 5mm (0.68±0.29) |
|  |  |  |  |  |  |  |  | **C**: 1mm (1.09±0.34), 3mm (1.08±0.47), 5mm (0.84±0.39); |
|  |  |  |  |  |  |  |  | **1PM**: 1 mm (1.23±0.4), 3mm (1.26±0.51), 5mm (1.16±0.49) |
|  |  |  |  |  |  |  |  | **2PM**: 1mm (1.63±0.62), 3mm (2.01±0.83), 5mm (1.99±0.92) |
| Zhang/2016 | *Implant Dent* | Case-control study | 239 (120 males, 119 female) | 45 ± 10.8 | CB MercuRay: 15 Ma, 120kV, FOV 4, voxel size 0.38 | Bone crest, middle root | 956 (CI, LI) | **CI**: P1 (1.21 ± 0.41), P2 (1.34 ± 0.44); |
|  |  |  |  |  |  |  |  | **LI**: P1 (1.27 ± 0.5), P2 (1.47 ± 0.46) |
| Zhang/2015 | *Implant Dent* | CS | 105 (69 males, 46 females) | 32.5 (20-48) | ProMax 3D Max: 82 kV, 8 mA, and 12 seconds | Crestal, middle root, apical | 630 (CI, LI, C) | **CI:** crestal (0.86±0.23), middle root (0.79±0.25), apical (0.95±0.39) |
|  |  |  |  |  |  |  |  | **LI:** crestal (0.9±0.29), middle root (0.61±0.25), apical (0.76±0.38) |
|  |  |  |  |  |  |  |  | **C:** crestal (0.98±0.31), middle root (0.65±0.35), apical (0.67±0.42) |
| Zhou/2014 | *J Biomed Res* | CS | 80 (33 males, 47 females) | 23.08 ± 6.3 (18-42) | New Torn VG: 110 kV, 5 mA, 0.25 mm slice, 36 seconds | 3 mm from CEJ, middle root, apical | 480 (CI, LI, C) | **CI:** 3 mm (0.98±0.32), middle root (0.96±0.24), apical (2.04±0.98) |
|  |  |  |  |  |  |  |  | **LI:** 3 mm (0.87±0.36), middle root (0.59±0.3), apical (2.07±1.04) |
|  |  |  |  |  |  |  |  | **C:** 3 mm (1.26±0.62), middle root (0.74±0.27), apical (1.65±0.66) |
