# Supplementary material for: Facial alveolar bone thickness and modifying factors of anterior maxillary teeth: a systematic review and meta-analysis of cone-beam computed tomography studies
Source: BMC Oral Health. 2021 Mar 22;21:143. doi: 10.1186/s12903-021-01495-2 (PMC7986564; doi:10.1186/s12903-021-01495-2)
Supplement: Supplementary file 3 — Additional file 3. Influence of age and sex subgroups on FAB thickness at different points from FBC and influence of the gingival phenotype on FAB thickness at different points. [file 12903_2021_1495_MOESM3_ESM.docx]

**Additional file 3.** Influence of age and gender subgroups upon FAB thickness at different points from FBC, and influence of gingival phenotype upon FAB thickness at different points.

|  | |  |  |  |  |  |  |  |  |  |
| --- | --- | --- | --- | --- | --- | --- | --- | --- | --- | --- |
| **FAB thickness from FBC** | | | | | | | | | | |
| **Tooth type** | **Reference point** | | **Covariable** | **N (teeth number)** | **WMD** | **SE** | **95% CI** | **I^2^** | **Q_H_ (p-value)** | **z (p-value)** |
| **CI** | 1mm | | Age | 5 (1544) | -0.04 | 0.04 | -0.11 0.04 | 82.70% | <0.001*** | 0.354 |
|  |  |  | Sex | 7 (1432) | 0 | 0.05 | -0.09 0.09 | 90.80% | <0.001*** | 0.879 |
|  | 3mm | | Age | 3 (933) | -0.05 | 0.05 | -0.14 0.04 | 69.30% | 0.038 | 0.308 |
|  |  |  | Sex | 3 (565) | 0 | 0.04 | -0.07 0.07 | 43.40% | 0.171 | 0.992 |
|  | 5mm | | Age | 4 (1020) | -0.07 | 0.04 | -0.14 -0.00 | 70.20% | 0.018 | **0.049*** |
|  |  |  | Sex | 5 (916) | -0.04 | 0.02 | -0.08 -0.00 | 0.0% | 0.747 | **0.034*** |
|  | 1mm | | Age | 5 (1435) | -0.05 | 0.05 | -0.14 0.05 | 82.20% | <0.001*** | 0.329 |
| **LI** |  | | Sex | 7 (1315) | -0.06 | 0.03 | -0.13 0.01 | 71.10% | 0.002** | **0.038*** |
|  | 3mm | | Age | 3 (807) | -0.06 | 0.06 | -0.19 0.06 | 68.80% | 0.04 | 0.321 |
|  |  | | Sex | 3 (439) | 0 | 0.05 | -0.09 0.09 | 45.90% | 0.158 | 0.941 |
|  | 5mm | | Age | 4 (830) | -0.06 | 0.05 | -0.16 0.04 | 76.40% | 0.005** | 0.239 |
|  |  | | Sex | 5 (726) | 0.01 | 0.03 | -0.04 0.06 | 0.00% | 0.846 | 0.64 |
| **C** | 1mm | | Age | 3 (780) | 0.01 | 0.04 | -0.06 0.08 | 0.00% | 0.917 | 0.735 |
|  |  | | Sex | 4 (645) | -0.08 | 0.03 | -0.14 0.03 | 23.90% | 0.268 | **0.004**** |
|  | 3mm | | Age | 3 (772) | -0.02 | 0.04 | -0.09 0.05 | 0.00% | 0.712 | 0.627 |
|  |  | | Sex | 3 (404) | -0.07 | 0.04 | -0.14 0.01 | 0.00% | 0.6 | 0.077 |
|  | 5mm | | Age | 3 (714) | -0.06 | 0.03 | -0.11 -0 | 0.00% | 0.563 | **0.049*** |
|  |  | | Sex | 4 (610) | -0.02 | 0.07 | -0.16 0.12 | 75.80% | 0.006** | 0.771 |
| **FAB thickness from CEJ** | | | | | | | | | | |
| **Tooth type** | **Reference point** | | **Covariable** | **N (teeth number)** | **WMD** | **SE** | **95% CI** | **I^2^** | **Q_H_ (p-value)** | **z (p-value)** |
| **CI** | 4 mm | | Gingival phenotype | 3 (417) | 0.35 | 0.09 | 0.17 0.54 | 90.90% | <0.001*** | **<0.001***** |
|  | 6 mm | |  | 3 (417) | 0.38 | 0.12 | 0.15 0.61 | 95.20% | <0.001*** | **0.001**** |
|  | 4 mm | | Gingival phenotype | 3 (390) | 0.5 | 0.13 | 0.24 0.77 | 90.00% | <0.001*** | **<0.001***** |
| **LI** | 6 mm | |  | 3 (399) | 0.54 | 0.13 | 0.29 0.80 | 89.70% | 0.001** | **<0.001***** |
| **C** | 4 mm | | Gingival phenotype | 3 (335) | 0.54 | 0.18 | 0.18 0.89 | 92.10% | <0.001*** | **0.003**** |
|  | 6 mm | |  | 3 (344) | 0.56 | 0.17 | 0.23 0.90 | 90.50% | <0.001*** | **0.001**** |
| \| **N, study number; WMD, weighted mean difference; SE, standard error; CI, confidence interval; I2, I-squared; QH, Cochran´s Q** \| \| --- \| \| ***p<0.05; **p<0.01; ***p<0.001** \| | | | | | | | | | | |
