# Supplementary material for: Facial alveolar bone thickness and modifying factors of anterior maxillary teeth: a systematic review and meta-analysis of cone-beam computed tomography studies
Source: BMC Oral Health. 2021 Mar 22;21:143. doi: 10.1186/s12903-021-01495-2 (PMC7986564; doi:10.1186/s12903-021-01495-2)
Supplement: Supplementary file 4 — Additional file 4. Measurement of FAB thickness with CEJ as reference point analyzed by meta-regression of the geographical study setting at CI, LI and C. [file 12903_2021_1495_MOESM4_ESM.docx]

**Additional file 4.** Measurement of FAB thickness with CEJ as reference point analyzed by meta-regression of the geographical study setting at CI, LI and C.

| **Tooth type** | **Reference point** | **N (teeth number)** | **Region** | **Beta** | **SE** | **95% CI** | **z (p-value)** | **R^2^** |
| --- | --- | --- | --- | --- | --- | --- | --- | --- |
|  |  |  |  |  |  |  |  |  |
| **CI** | 4 mm | 7 (1618) | Asia (ref.) |  |  |  | 0.351 | 4.57% |
|  |  |  | Europe | -0.26 | 0.21 | -0.68 0.16 | 0.222 |  |
|  |  |  | America | -0.33 | 0.27 | -0.86 0.19 | 0.216 |  |
|  |  |  | Africa | -0.39 | 0.27 | -0.92 0.14 | 0.145 |  |
|  | middle root | 7 (1847) | Asia (ref.) |  |  |  | 0.215 | 16.80% |
|  |  |  | Europe | -0.14 | 0.1 | -0.34 0.06 | 0.172 |  |
|  |  |  | Africa | -0.18 | 0.13 | -0.43 0.07 | 0.159 |  |
| **LI** | 4 mm | 7 (1606) | Asia (ref.) |  |  |  | 0.763 | 0.00% |
|  |  |  | Europe | -0.02 | 0.24 | -0.49 0.44 | 0.917 |  |
|  |  |  | America | -0.22 | 0.29 | -0.79 0.36 | 0.459 |  |
|  |  |  | Africa | -0.26 | 0.29 | -0.84 0.32 | 0.382 |  |
|  | middle root | 6 (1562) | Asia (ref.) |  |  |  | 0.78 | 0.00% |
|  |  |  | Europe | 0.08 | 0.13 | -0.18 0.33 | 0.564 |  |
|  |  |  | Africa | -0.03 | 0.16 | -0.35 0.28 | 0.835 |  |
| **C** | 4 mm | 7 (1544) | Asia (ref.) |  |  |  | 0.625 | 0.00% |
|  |  |  | Europe | -0.07 | 0.23 | -0.53 0.39 | 0.758 |  |
|  |  |  | America | -0.31 | 0.29 | -0.88 0.27 | 0.293 |  |
|  |  |  | Africa | -0.3 | 0.29 | -0.87 0.28 | 0.311 |  |
|  | middle root | 6 (1555) | Asia (ref.) |  |  |  | 0.055 | 53.10% |
|  |  |  | Europe | -0.06 | 0.07 | -0.19 0.07 | 0.371 |  |
|  |  |  | Africa | -0.18 | 0.07 | -0.32 -0.03 | **0.017*** |  |
| **N, study number; SE, standard error; CI, confidence interval; I2, I-squared; QH, Cochran´s Q; R2, R-squared** | | | | | | | | |
| ***p<0,05; **p<0,01; ***p<0,001** | | | | | | | | |
