# Supplementary material for: Facial alveolar bone thickness and modifying factors of anterior maxillary teeth: a systematic review and meta-analysis of cone-beam computed tomography studies
Source: BMC Oral Health. 2021 Mar 22;21:143. doi: 10.1186/s12903-021-01495-2 (PMC7986564; doi:10.1186/s12903-021-01495-2)
Supplement: Supplementary file 5 — Additional file 5. Measurement of CEJ-FBC distance analyzed by meta-regression of study geographical setting CI, LI and C. [file 12903_2021_1495_MOESM5_ESM.docx]

**Additional file 5.** Measurement of CEJ-FBC distance analyzed by meta-regression of the geographical study setting at CI, LI and C.

| **Tooth type** | **N (teeth number)** | **Region** | **Beta** | **SE** | **95% CI** | **z (p-value)** | **R^2^** |
| --- | --- | --- | --- | --- | --- | --- | --- |
|  |  |  |  |  |  |  |  |
| **CI** | 14 (3024) | Asia (ref.) |  |  |  | 0.218 | 12.30% |
|  |  | Europe | 0.36 | 0.27 | -0.16 0.88 | 0.174 |  |
|  |  | America | 0.53 | 0.3 | -0.06 1.12 | 0.08 |  |
|  |  | Africa | -0.10 | 0,41 | -0.90 0.70 | 0.806 |  |
| **LI** | 13 (2685) | Asia (ref.) |  |  |  | 0.252 | 9.26% |
|  |  | Europe |  |  |  |  |  |
|  |  | America | -0.59 | 0.36 | -1.63 0.10 | 0.103 |  |
|  |  | Africa | -0.34 | 0.45 | -1.22 0.54 | 0.453 |  |
| **C** | 10 (2111) | Asia (ref.) |  |  |  | **<0.001***** | 73.50% |
|  |  | Europe | 0.54 | 0.18 | 0.18 0.90 | **0.003**** |  |
|  |  | America | 0.95 | 0.2 | 0.56 1.34 | **<0.001***** |  |
| **N, study number; SE, standard error; CI, confidence interval; I2, I-squared; QH, Cochran´s Q; R2, R-squared** | | | | | | | |
| ***p<0,05; **p<0,01; ***p<0,001** | | | | | | | |

The study geographical setting in C explains a great part of heterogeneity (R^2^=74%), studies from Asia reported shorter CEJ-FBC distance than those from Europe (beta=0.18) (p=0.003) and America (beta=0.95) (p<0.001).

At CI, the propensity for risk of confounding and selection bias explains the 35 and 31% of whole heterogeneity, generating an overestimation of CEJ-FBC distance of 0.91 to 1.23 mm than those with low risk of bias. The geographical localization explains 18% of whole heterogeneity in this data subset.

**Propensity for risk of bias on CEJ-FBC distance**

At LI the propensity for risk of confounding and selection bias explains the 43% and 32% of whole heterogeneity, generating an overestimation of CEJ-FBC distance of 0.78 to 0.99 mm than those with low risk of bias, respectively.

At C The propensity for risk of confounding showed a marginal trend towards significativity (p=0.060) and selection bias explains the 31% (p=0.053) of whole heterogeneity, generating an overestimation of CEJ-FAB distance of 0.85 to 1.10 mm than those with low risk of bias, respectively.
