# Supplementary material for: Facial alveolar bone thickness and modifying factors of anterior maxillary teeth: a systematic review and meta-analysis of cone-beam computed tomography studies
Source: BMC Oral Health. 2021 Mar 22;21:143. doi: 10.1186/s12903-021-01495-2 (PMC7986564; doi:10.1186/s12903-021-01495-2)
Supplement: Supplementary file 6 — Additional file 6. Supplementary Table S1. Assessment of the certainty of evidence by GRADE tool for FAB thickness considering FBC as anatomical reference. Supplementary Table S2. Assessment of the certainty of evidence by GRADE tool for FAB thickness considering CEJ as anatomical reference. Supplementary Table S3. Assessment of the certainty of evidence by GRADE tool for CEJ-FBC distance. [file 12903_2021_1495_MOESM6_ESM.docx]

**Additional file 6:** Assessment of the certainty of evidence by GRADE tool.

**Supplementary Table 1 “Table S1”**: Assessment of the certainty of evidence using the GRADE tool for FAB thickness, taking FBC as anatomical reference.

| **Summary of findings:** | | | | | | |
| --- | --- | --- | --- | --- | --- | --- |
| **CBCT for Facial Alveolar Bone thickness of anterior maxillary teeth considering FBC as anatomical reference** | | | | | | |
| **Patient or population**: Facial Alveolar Bone thickness of anterior maxilla teeth  **Setting**: Healthy patients >18 years without absence of anterior teeth  **Intervention**: CBCT  **Comparison**: None | | | | | | |
| Outcomes | **Anticipated absolute effects^*^** (95% CI) | | Relative effect (95% CI) | № of teeth included (studies) | Certainty of the evidence (GRADE) | Comments |
|  | **Risk with None** | **Risk with CBCT** |  |  |  |  |
| Central Incisors FAB thickness at 1 mm from FBC | - | WM **0.9 mm higher** (0.78 higher to 1.01 higher) | - | 3038 (14 observational studies) | ⨁⨁◯◯ LOW ^a,b,c,d^ | The evidence is downgraded by two levels due to the presence of selection and confounding bias inherent to the observational study design. The evidence is downgraded one level both by inconsistency and imprecision. Evidence is rating up by the plausibility of the spurious effect by confounders. |
| Central Incisors FAB thickness at 3 mm from FBC | - | WM **0.98 mm higher** (0.77 higher to 1.19 higher) | - | 1369 (7 observational studies) | ⨁⨁⨁◯ MODERATE ^a,c,d,e^ | The evidence is downgraded by two levels due to the presence of selection and confounding bias inherent to the observational study design. The evidence is downgraded one level by imprecision. Evidence is rating up by the plausibility of the spurious effect by confounders. |
| Central Incisors FAB thickness at 5 mm from FBC | - | WM **0.86 mm higher** (0.7 higher to 1.01 higher) | - | 1654 (8 observational studies) | ⨁⨁⨁◯ MODERATE ^a,c,d,e^ | The evidence is downgraded by two levels due to the presence of selection and confounding bias inherent to the observational study design. The evidence is downgraded one level by imprecision. Evidence is rating up by the plausibility of the spurious effect by confounders. |
| Lateral Incisors FAB thickness at 1 mm from FBC | - | WM **0.89 mm higher** (0.77 higher to 1 higher) | - | 2721 (13 observational studies) | ⨁⨁⨁◯ MODERATE ^a,c,e^ | The evidence is downgraded by two levels due to the presence of selection and confounding bias inherent to the observational study design. Evidence is rating up by the plausibility of the spurious effect by confounders. |
| Lateral Incisors FAB thickness at 3 mm from FBC | - | WM **1.03 mm higher** (0.74 higher to 1.32 higher) | - | 957 (6 observational studies) | ⨁⨁⨁◯ MODERATE ^a,c,f^ | The evidence is downgraded by two levels due to the presence of selection and confounding bias inherent to the observational study design. Evidence is rating up by the plausibility of the spurious effect by confounders. |
| Lateral Incisors FAB thickness at 5 mm from FBC | - | WM **0.75 mm higher** (0.54 higher to 0.96 higher) | - | 1266 (7 observational studies) | ⨁⨁⨁◯ MODERATE ^a,c,f^ | The evidence is downgraded by two levels due to the presence of selection and confounding bias inherent to the observational study design. Evidence is rating up by the plausibility of the spurious effect by confounders. |
| Canines FAB thickness at 1 mm from FBC | - | WM **0.91 mm higher** (0.78 higher to 1.05 higher) | - | 1652 (9 observational studies) | ⨁⨁⨁◯ MODERATE ^a,c,f^ | The evidence is downgraded by two levels due to the presence of selection and confounding bias inherent to the observational study design. Evidence is rating up by the plausibility of the spurious effect by confounders. |
| Canines FAB thickness at 3 mm from FBC | - | WM **1.05 mm higher** (0.81 higher to 1.29 higher) | - | 1006 (6 observational studies) | ⨁⨁⨁◯ MODERATE ^a,c,f^ | The evidence is downgraded by two levels due to the presence of selection and confounding bias inherent to the observational study design. Evidence is rating up by the plausibility of the spurious effect by confounders. |
| Canines FAB thickness at 5 mm from FBC | - | WM **0.84 mm higher** (0.62 higher to 1.05 higher) | - | 1150 (6 observational studies) | ⨁⨁⨁◯ MODERATE ^a,c,f^ | The evidence is downgraded by two levels due to the presence of selection and confounding bias inherent to the observational study design. Evidence is rating up by the plausibility of the spurious effect by confounders. |
| 1PM FAB thickness at 1 mm from FBC | - | WM **1.17 mm higher** (0.98 higher to 1.38 higher) | - | 491 (4 observational studies) | ⨁⨁⨁◯ MODERATE ^a,c,g,h^ | The evidence is downgraded by two levels due to the presence of selection and confounding bias inherent to the observational study design. Evidence is rating up by the plausibility of the spurious effect by confounders. |
| 1PM FAB thickness at 3 mm from FBC | - | WM **1.26 mm higher** (0.76 higher to 1.76 higher) | - | 461 (3 observational studies) | ⨁⨁◯◯ LOW ^a,c,d,g^ | The evidence is downgraded by two levels due to the presence of selection and confounding bias inherent to the observational study design. The evidence is downgraded one level by imprecision. Evidence is rating up by the plausibility of the spurious effect by confounders. |
| 1PM FAB thickness at 5 mm from FBC | - | WM **1.09 mm higher** (0.65 higher to 1.52 higher) | - | 432 (3 observational studies) | ⨁⨁◯◯ LOW ^a,c,d,g^ | The evidence is downgraded by two levels due to the presence of selection and confounding bias inherent to the observational study design. The evidence is downgraded one level by imprecision. Evidence is rating up by the plausibility of the spurious effect by confounders. |
| 2PM FAB thickness at 1 mm from FBC | - | WM **1.57 mm higher** (1.42 higher to 1.71 higher) | - | 534 (4 observational studies) | ⨁⨁⨁◯ MODERATE ^a,c,g,h^ | The evidence is downgraded by two levels due to the presence of selection and confounding bias inherent to the observational study design. Evidence is rating up by the plausibility of the spurious effect by confounders. |
| 2PM FAB thickness at 3 mm from FBC | - | WM **1.96 mm higher** (1.38 higher to 2.53 higher) | - | 508 (3 observational studies) | ⨁⨁◯◯ LOW ^a,c,d,g^ | The evidence is downgraded by two levels due to the presence of selection and confounding bias inherent to the observational study design. The evidence is downgraded one level by imprecision. Evidence is rating up by the plausibility of the spurious effect by confounders. |
| 2PM FAB thickness at 5 mm from FBC | - | WM **1.82 mm higher** (1.15 higher to 2.49 higher) | - | 508 (3 observational studies) | ⨁⨁◯◯ LOW ^a,c,d,g^ | The evidence is downgraded by two levels due to the presence of selection and confounding bias inherent to the observational study design. The evidence is downgraded one level by imprecision. Evidence is rating up by the plausibility of the spurious effect by confounders. |
| ***The risk in the intervention group** (and its 95% confidence interval) is based on the assumed risk in the comparison group and the **relative effect** of the intervention (and its 95% CI).   **CI:** Confidence interval **CBCT:** Cone beam computed tomography **FAB:** Facial alveolar bone **FBC:** Facial bone crest | | | | | | |
| **GRADE Working Group grades of evidence** **High certainty:** We are very confident that the true effect lies close to that of the estimate of the effect **Moderate certainty:** We are moderately confident in the effect estimate: The true effect is likely to be close to the estimate of the effect, but there is a possibility that it is substantially different **Low certainty:** Our confidence in the effect estimate is limited: The true effect may be substantially different from the estimate of the effect **Very low certainty:** We have very little confidence in the effect estimate: The true effect is likely to be substantially different from the estimate of effect | | | | | | |

#### Explanations

a. The evidence is downgraded by two levels due to the presence of selection and confounding bias inherent to the observational study design.

b. The high heterogeneity context was explained in less extent (16%) by effect modifier (geographic latitude).

c. Indirectness was expected due to biological and social factors of the patients, and by differences on exposure "CBCT features". The geographic location and the isotropic voxel size are considered as effect modifiers. However, geographic latitude explains heterogeneity and voxel size did not show statistical significance as a covariate. The evidence is not downgraded by indirectness.

d. The lower and upper bounds of the 95% confidence interval do not include the zero. Maximum error measurement of about +-0.20 mm is established as a threshold for measurement accuracy according to a previous report [62]. Meta-analytic standard deviations surpassing in a tenth, the error point, are considered imprecise. The evidence is downgraded one level by imprecision.

e. The high heterogeneity context was explained in a moderate extent by effect modifier (geographic latitude). The high heterogeneity is of questionable importance. There is a quantitative interaction of measurements among studies.

f. The high heterogeneity context was explained in a greater extent by effect modifier (geographic latitude). The high heterogeneity is of questionable importance. There is a quantitative interaction of measurements among studies.

g. The high heterogeneity is of questionable importance. There is a quantitative interaction of measurements among studies.

h. The lower and upper bounds of the 95% confidence interval do not include the zero. Maximum error measurement of about +-0.20 mm is established as a threshold for measurement accuracy according to a previous report [62]. Meta-analytic standard deviations surpassing in a tenth, the error point, are considered imprecise. The evidence is not downgraded by imprecision.

**Supplementary Table 2 “Table S2”**: Assessment of the certainty of evidence using the GRADE tool for FAB thickness, taking CEJ as anatomical reference.

| **Summary of findings:** | | | | | | |
| --- | --- | --- | --- | --- | --- | --- |
| **CBCT for Facial Alveolar Bone thickness of anterior maxillary teeth considering CEJ as anatomical reference** | | | | | | |
| **Patient or population**: Facial Alveolar Bone thickness of anterior maxillary teeth  **Setting**: Healthy patients >18 years without absence of anterior teeth  **Intervention**: CBCT  **Comparison**: [none] | | | | | | |
| Outcomes | **Anticipated absolute effects^*^** (95% CI) | | Relative effect (95% CI) | № of teeth included  (studies) | Certainty of the evidence (GRADE) | Comments |
|  | **Risk with [none]** | **Risk with CBCT** |  |  |  |  |
| Central Incisors FAB thickness at 4 mm from CEJ | - | WM **0.79 MM higher** (0.61 higher to 0.97 higher) | - | 1618 (7 observational studies) | ⨁⨁◯◯ LOW ^a,b,c,d,e^ | Evidence is rating down three levels due to an extremely serious risk of bias that may affect the confidence of the results. Evidence is rating up by the plausibility of the spurious effect by confounders. |
| Central Incisors FAB thickness at 6 mm from CEJ | - | WM **0.9 mm higher** (0.71 higher to 1.09 higher) | - | 769 (4 observational studies) | ⨁⨁◯◯ LOW ^a,b,c,d,e^ | Evidence is rating down three levels due to an extremely serious risk of bias that may affect the confidence of the results. Evidence is rating up by the plausibility of the spurious effect by confounders. |
| Central Incisors FAB thickness at middle root from CEJ | - | WM **0.8 mm higher** (0.7 higher to 0.89 higher) | - | 1847 (7 observational studies) | ⨁⨁◯◯ LOW ^a,b,c,d,e^ | Evidence is rating down three levels due to an extremely serious risk of bias that may affect the confidence of the results. Evidence is rating up by the plausibility of the spurious effect by confounders. |
| Lateral Incisors FAB thickness at 4 mm from CEJ | - | WM **0.76 mm higher** (0.6 higher to 0.92 higher) | - | 1606 (7 observational studies) | ⨁⨁◯◯ LOW ^a,b,d,e,f^ | Evidence is rating down three levels due to an extremely serious risk of bias that may affect the confidence of the results. Evidence is rating up by the plausibility of the spurious effect by confounders. |
| Lateral Incisors FAB thickness at 6 mm from CEJ | - | WM **0.79 mm higher** (0.65 higher to 0.93 higher) | - | 423 (3 observational studies) | ⨁⨁⨁◯ MODERATE ^a,d,e,f^ | The evidence is downgraded by two levels due to the presence of moderate risk of selection and confounding bias inherent to the observational study design. Evidence is rating up by the plausibility of the spurious effect by confounders. |
| Lateral Incisors FAB thickness at middle root from CEJ | - | WM **0.65 mm higher** (0.56 higher to 0.73 higher) | - | 1652 (6 observational studies) | ⨁⨁⨁◯ MODERATE ^a,c,d,e^ | The evidence is downgraded by two levels due to the presence of moderate risk of selection and confounding bias inherent to the observational study design. Evidence is rating up by the plausibility of the spurious effect by confounders. |
| Canines FAB thickness at 4 mm from CEJ | - | WM **0.7 mm higher** (0.53 higher to 0.87 higher) | - | 1544 (7 observational studies) | ⨁⨁◯◯ LOW ^a,b,d,e,f^ | Evidence is rating down three levels due to an extremely serious risk of bias that may affect the confidence of the results. Evidence is rating up by the plausibility of the spurious effect by confounders. Evidence is rating up by the plausibility of the spurious effect by confounders. |
| Canines FAB thickness at 6 mm from CEJ | - | WM **0.72 mm higher** (0.55 higher to 0.89 higher) | - | 368 (3 observational studies) | ⨁⨁◯◯ LOW ^a,b,c,d,e^ | Evidence is rating down three levels due to an extremely serious risk of bias that may affect the confidence of the results. Evidence is rating up by the plausibility of the spurious effect by confounders. |
| Canines FAB thickness at middle root from CEJ | - | WM **0.65 mm higher** (0.58 higher to 0.73 higher) | - | 1555 (6 observational studies) | ⨁⨁⨁◯ MODERATE ^a,d,e,g^ | The evidence is downgraded by two levels due to the presence of moderate risk of selection and confounding bias inherent to the observational study design. Evidence is downgraded by one additional level due to the presence of a study with a serious risk of selection bias. Evidence is rating up by the plausibility of the spurious effect by confounders. |
| First Premolars FAB thickness at 4 mm from CEJ | - | WM **1.04 mm higher** (0.74 higher to 1.33 higher) | - | 374 (3 observational studies) | ⨁⨁◯◯ LOW ^a,c,d,e,h^ | The evidence is downgraded by two levels due to the presence of moderate risk of selection and confounding bias inherent to the observational study design. Evidence is downgraded by one additional level due to the presence of a study with a serious risk of selection bias. Evidence is rating up by the plausibility of the spurious effect by confounders. |
| First Premolars FAB thickness at middle root from CEJ | - | WM **0.97 mm higher** (0.51 higher to 1.43 higher) | - | 680 (2 observational studies) | ⨁⨁◯◯ LOW ^a,d,f,i^ | The evidence is downgraded by two levels due to the presence of moderate risk of selection and confounding bias inherent to the observational study design. The evidence is rating down one level by imprecision. Evidence is rating up by the plausibility of the spurious effect by confounders. |
| Second Premolars FAB thickness at 4 mm from CEJ | - | WM **1.53 mm higher** (1.17 higher to 1.88 higher) | - | 664 (2 observational studies) | ⨁⨁◯◯ LOW ^a,d,f,i^ | The evidence is downgraded by two levels due to the presence of moderate risk of selection and confounding bias inherent to the observational study design. The evidence is rating down one level by imprecision. Evidence is rating up by the plausibility of the spurious effect by confounders. |
| ***The risk in the intervention group** (and its 95% confidence interval) is based on the assumed risk in the comparison group and the **relative effect** of the intervention (and its 95% CI).   **CI:** Confidence interval | | | | | | |
| **GRADE Working Group grades of evidence** **High certainty:** We are very confident that the true effect lies close to that of the estimate of the effect **Moderate certainty:** We are moderately confident in the effect estimate: The true effect is likely to be close to the estimate of the effect, but there is a possibility that it is substantially different **Low certainty:** Our confidence in the effect estimate is limited: The true effect may be substantially different from the estimate of the effect **Very low certainty:** We have very little confidence in the effect estimate: The true effect is likely to be substantially different from the estimate of effect | | | | | | |

#### Explanations

a. The evidence is downgraded by two levels due to the presence of moderate risk of selection and confounding bias inherent to the observational study design.

b. Evidence is downgraded by one additional level due to the presence of a study with a serious risk of confounding and selection bias.

c. The high heterogeneity context was explained in less extent by effect modifier (geographic latitude). The high heterogeneity is of questionable importance. There is a quantitative interaction of measurements among studies.

d. Indirectness was expected due to biological and social factors of the patients, and by differences on exposure "CBCT features". The geographic location and the isotropic voxel size are considered as effect modifiers. However, geographic latitude explains heterogeneity and voxel size did not show statistical significance as a covariate. The evidence is not downgraded by indirectness.

e. The lower and upper bounds of the 95% confidence interval do not include the zero. Maximum error measurement of about +-0.20 mm is established as a threshold for measurement accuracy according to a previous report [62]. Meta-analytic standard deviations surpassing in a tenth, the error point, are considered imprecise. The evidence is not downgraded by imprecision.

f. The high heterogeneity is of questionable importance. There is a quantitative interaction of measurements among studies.

g. The high heterogeneity context was explained in great extent by effect modifier (geographic latitude). The high heterogeneity is of questionable importance. There is a quantitative interaction of measurements among studies.

h. Evidence is downgraded by one additional level due to the presence of a study with a serious risk of selection bias.

i. The lower and upper bounds of the 95% confidence interval do not include the zero. Maximum error measurement of about +-0.20 mm is established as a threshold for measurement accuracy according to a previous report [62]. Meta-analytic standard deviations surpassing in a tenth, the error point, are considered imprecise. The evidence is rating down one level by imprecision.

**Supplementary Table 3 “Table S3”**: Assessment of the certainty of evidence using the GRADE tool for CEJ-FBC distance.

| **Summary of findings:** | | | | | | |
| --- | --- | --- | --- | --- | --- | --- |
| **CBCT compared to [none] for CEJ to FBC crest distance in mm** | | | | | | |
| **Patient or population**: CEJ to FBC crest distance in mm  **Setting**: Healthy patients >18 years without absence of anterior teeth  **Intervention**: CBCT  **Comparison**: [none] | | | | | | |
| Outcomes | **Anticipated absolute effects^*^** (95% CI) | | Relative effect (95% CI) | № of teeth included (studies) | Certainty of the evidence (GRADE) | Comments |
|  | **Risk with [none]** | **Risk with CBCT** |  |  |  |  |
| CEJ to FBC distance at Central Incisors | - | WM **2.24 mm higher** (2 higher to 2.48 higher) | - | 3024 (14 observational studies) | ⨁⨁⨁◯ MODERATE ^a,b,c,d,e,f^ | The evidence is downgraded by two levels due to the presence of moderate risk of selection and confounding bias inherent to the observational study design. Evidence is rating up by the plausibility of the spurious effect by confounders. |
| Effect of gender on the CEJ to FBC distance at Central Incisors | - | WMD **0.32 mm lower** (0.53 lower to 0.12 lower) | - | 1032 (5 observational studies) | ⨁⨁⨁◯ MODERATE ^a,e,f,g^ | The evidence is downgraded by two levels due to the presence of moderate risk of selection and confounding bias inherent to the observational study design. Evidence is rating up by the plausibility of the spurious effect by confounders. |
| Effect of age on the CEJ to FBC distance at Central Incisors | - | WMD **0.83 mm higher** (0.28 higher to 1.38 higher) | - | 1798 (6 observational studies) | ⨁⨁⨁◯ MODERATE ^a,e,h,i^ | The evidence is downgraded by two levels due to the presence of moderate risk of selection and confounding bias inherent to the observational study design. Evidence is rating up by the plausibility of the spurious effect by confounders. |
| CEJ to FBC distance at Lateral Incisors | - | WM **2.35 mm higher** (2.13 higher to 2.57 higher) | - | 2685 (13 observational studies) | ⨁⨁⨁◯ MODERATE ^a,c,d,e,f^ | The evidence is downgraded by two levels due to the presence of moderate risk of selection and confounding bias inherent to the observational study design. Evidence is rating up by the plausibility of the spurious effect by confounders. |
| Effect of gender on the CEJ to FBC distance at Lateral Incisors | - | WMD **0.19 mm lower** (0.46 lower to 0.09 higher) | - | 722 (4 observational studies) | ⨁⨁◯◯ LOW ^a,e,h,j^ | The evidence is downgraded by two levels due to the presence of moderate risk of selection and confounding bias inherent to the observational study design. Evidence is downgraded one level by imprecision. Evidence is rating up by the plausibility of the spurious effect by confounders. |
| Effect of age on the CEJ to FBC distance at Lateral Incisors | - | WMD **0.82 mm higher** (0.37 higher to 1.27 higher) | - | 1690 (6 observational studies) | ⨁⨁⨁◯ MODERATE ^a,e,i,k^ | The evidence is downgraded by two levels due to the presence of moderate risk of selection and confounding bias inherent to the observational study design. Evidence is rating up by the plausibility of the spurious effect by confounders. |
| CEJ to FBC distance at Canines | - | WM **2.53 mm higher** (2.24 higher to 2.81 higher) | - | 2111 (10 observational studies) | ⨁⨁⨁◯ MODERATE ^a,c,d,e,f^ | The evidence is downgraded by two levels due to the presence of moderate risk of selection and confounding bias inherent to the observational study design. Evidence is rating up by the plausibility of the spurious effect by confounders. |
| Effect of gender on the CEJ to FBC distance at Canines | - | WMD **0.01 mm higher** (0.61 lower to 0.63 higher) | - | 545 (3 observational studies) | ⨁⨁◯◯ LOW ^a,c,d,e,j^ | The evidence is downgraded by two levels due to the presence of moderate risk of selection and confounding bias inherent to the observational study design. Evidence is downgraded one level by imprecision. Evidence is rating up by the plausibility of the spurious effect by confounders. |
| Effect of age on the CEJ to FBC distance at Canines | - | WMD **0.75 mm higher** (0.27 higher to 1.23 higher) | - | 1513 (5 observational studies) | ⨁⨁⨁◯ MODERATE ^a,e,h,i^ | The evidence is downgraded by two levels due to the presence of moderate risk of selection and confounding bias inherent to the observational study design. Evidence is rating up by the plausibility of the spurious effect by confounders. |
| CEJ to FBC distance at First Premolars | - | WM **2.33 mm higher** (2.16 higher to 2.49 higher) | - | 907 (3 observational studies) | ⨁⨁⨁◯ MODERATE ^a,e,f,h^ | The evidence is downgraded by two levels due to the presence of moderate risk of selection and confounding bias inherent to the observational study design. Evidence is rating up by the plausibility of the spurious effect by confounders. |
| CEJ to FBC distance at Second Premolars | - | WM **2.03 mm higher** (1.81 higher to 2.22 higher) | - | 952 (3 observational studies) | ⨁⨁⨁◯ MODERATE ^a,e,f,h^ | The evidence is downgraded by two levels due to the presence of moderate risk of selection and confounding bias inherent to the observational study design. Evidence is rating up by the plausibility of the spurious effect by confounders. |
| ***The risk in the intervention group** (and its 95% confidence interval) is based on the assumed risk in the comparison group and the **relative effect** of the intervention (and its 95% CI).   **CI:** Confidence interval | | | | | | |
| **GRADE Working Group grades of evidence** **High certainty:** We are very confident that the true effect lies close to that of the estimate of the effect **Moderate certainty:** We are moderately confident in the effect estimate: The true effect is likely to be close to the estimate of the effect, but there is a possibility that it is substantially different **Low certainty:** Our confidence in the effect estimate is limited: The true effect may be substantially different from the estimate of the effect **Very low certainty:** We have very little confidence in the effect estimate: The true effect is likely to be substantially different from the estimate of effect | | | | | | |

#### Explanations

a. The evidence is downgraded by two levels due to the presence of moderate risk of selection and confounding bias inherent to the observational study design.

b. Evidence is downgraded by one additional level due to the presence of a study with a serious risk of confounding and selection bias.

c. The high heterogeneity context was explained in great extent by effect modifier (geographic latitude). The high heterogeneity is of questionable importance. There is a quantitative interaction of measurements among studies.

d. The meta-regression analysis showed that propensity for serious risk of confounding and selection bias tend to overestimate the FBC-CEJ distance.

e. Indirectness was expected due to biological and social factors of the patients, and by differences on exposure "CBCT features". The geographic location, the isotropic voxel size, smoking and accounting for periodontal disease as inclusion criterion are considered as effect modifiers. However, geographic latitude explains heterogeneity; Also the other covariates did not show statistical significance. The evidence is not downgraded by indirectness.

f. The lower and upper bounds of the 95% confidence interval do not include the zero. Maximum error measurement of about +-0.20 mm is established as a threshold for measurement accuracy according to a previous report [62]. Meta-analytic standard deviations surpassing in a tenth, the error point, are considered imprecise. The evidence is not downgraded by imprecision.

g. The moderate heterogeneity context was explained in less extent by effect modifier (geographic latitude). The heterogeneity is of questionable importance. There is a quantitative interaction of measurements among studies.

h. The high heterogeneity is of questionable importance. There is a quantitative interaction of measurements among studies.

i. The lower and upper bounds of the 95% confidence interval do not include the zero. The evidence is not downgraded by imprecision.

j. The upper bound of the 95% confidence interval crossed the no effect—the evidence not downgraded one level by imprecision.

k. The high heterogeneity context was explained in less extent by effect modifier (geographic latitude). The heterogeneity is of questionable importance. There is a quantitative interaction of measurements among studies.
